# Do DEEPER ROOTING 1 Homologs Regulate the Lateral Root Slope Angle in Cucumber (*Cucumis sativus*)?

**DOI:** 10.3390/ijms25041975

**Published:** 2024-02-06

**Authors:** Alexey S. Kiryushkin, Elena L. Ilina, Tatyana Y. Kiikova, Katharina Pawlowski, Kirill N. Demchenko

**Affiliations:** 1 Laboratory of Cellular and Molecular Mechanisms of Plant Development, Komarov Botanical Institute, Russian Academy of Sciences, 197022 Saint Petersburg, Russia; akiryushkin@binran.ru (A.S.K.); eilina@binran.ru (E.L.I.);; 2 Department of Ecology, Environment and Plant Sciences, Stockholm University, 10691 Stockholm, Sweden

**Keywords:** CRISPR/Cas9, cucumber, *Cucumis sativus*, DEEPER ROOTING 1, DRO1, genome editing, IGT, lateral root, LAZY, root branching, root meristem, root system architecture

## Abstract

The architecture of the root system is fundamental to plant productivity. The rate of root growth, the density of lateral roots, and the spatial structure of lateral and adventitious roots determine the developmental plasticity of the root system in response to changes in environmental conditions. One of the genes involved in the regulation of the slope angle of lateral roots is *DEEPER ROOTING 1* (*DRO1*). Its orthologs and paralogs have been identified in rice, Arabidopsis, and several other species. However, nothing is known about the formation of the slope angle of lateral roots in species with the initiation of lateral root primordia within the parental root meristem. To address this knowledge gap, we identified orthologs and paralogs of the *DRO1* gene in cucumber (*Cucumis sativus*) using a phylogenetic analysis of IGT protein family members. Differences in the transcriptional response of *CsDRO1*, *CsDRO1-LIKE1* (*CsDRO1L1*), and *CsDRO1-LIKE2* (*CsDRO1L2)* to exogenous auxin were analyzed. The results showed that only *CsDRO1L1* is auxin-responsive. An analysis of promoter–reporter fusions demonstrated that the *CsDRO1*, *CsDRO1L1*, and *CsDRO1L2* genes were expressed in the meristem in cell files of the central cylinder, endodermis, and cortex; the three genes displayed different expression patterns in cucumber roots with only partial overlap. A knockout of individual *CsDRO1*, *CsDRO1L1*, and *CsDRO1L2* genes was performed via CRISPR/Cas9 gene editing. Our study suggests that the knockout of individual genes does not affect the slope angle formation during lateral root primordia development in the cucumber parental root.

## 1. Introduction

Root system architecture is determined by several morphological parameters like the growth rate of parental and lateral roots, the lateral root density, and the growth angle of adventitious or lateral roots [1,2,3]. Two of these parameters, growth rate and density, have been studied in more detail than the formation of the lateral root growth angle. The regulation of growth rate and density is the result of a coordinated interplay between many molecular actors, like phytohormones, numerous genes, and nutrient availability [2,4,5,6]. In comparison, our understanding of the growth angle formation of lateral or adventitious roots is superficial.

A gene named *DEEPER ROOTING 1* (*OsDRO1*)/*NEGATIVE GRAVITROPISM 3* (*OsNGR3*) was identified as the main regulator of rooting depth through a determination of the growth angle in rice (*Oryza sativa*) adventitious roots [7]. A single-nucleotide substitution in *OsDRO1* resulted in a premature stop codon, a consequent lack of a full-length protein, and the development of a shallow root system. *OsDRO1* was expressed in the meristem of parental and lateral roots. Under gravitropic stimulation, the zone of *OsDRO1* expression shifted to the outer side of the distal elongation zone, where the DRO1 protein probably stimulated the elongation of cells mediating the gravitropic response [7].

*OsDRO1* expression is negatively regulated by auxin. The uneven distribution of the *Os*DRO1 protein is due to auxin-mediated inhibition of *OsDRO1* expression on the inner side of the adventitious root [7]. The *OsDRO1* paralog *DRO1-like1* (*OsDRL1*)/*quantitative trait locus for SOIL SURFACE ROOTING1* (*OsqSOR1*)/*OsNGR2* was identified [8]. The expression of *OsDRL1*, like that of *OsDRO1*, is negatively regulated by auxin. Mutations of *OsDRL1*, as well as of *OsDRO1*, result in the formation of a shallow root system. A phylogenetic analysis of rice protein sequences also showed that besides *Os*DRO1 and *Os*DRL1, rice contains two others DRO1 homologs (*Os*DRL2 and *Os*DRL3/*Os*NGR1) with unknown functions [8].

Orthologs of *OsDRO1—AtDRO1*/*AtNGR2*/*AtLAZY4*, *AtDRO2*/*AtNGR3*/*AtLAZY3*, and *AtDRO3*/*AtNGR1*/*AtLAZY2*—have been identified in Arabidopsis [9,10]. Knockout mutants of the *AtDRO1* gene exhibit a wider growth angle of the lateral roots [9]. Also, a *dro3* (*lazy2*) mutant, as well as a *dro1dro3* (*lazy4lazy2*) double mutant, but not a *dro2* (*lazy3*) single mutant, exhibit a wider lateral root growth angle [10]. In summary, *AtDRO1* (*AtLAZY4*) and *AtDRO3* (*AtLAZY2*), but not *AtDRO2* (*AtLAZY3*), regulate the lateral root angle. Notably, the heterologous expression of potato *StDRO1* in Arabidopsis *dro1* single mutants partially rescues the gravitropic response of Arabidopsis lateral roots [11], which proves that *StDRO1*, like *AtDRO1*, is involved in the root gravitropic reaction.

A comparison of the amino acid sequences of DRO1-, LAZY1-, and TILLER ANGLE CONTROL1 (TAC1)-like proteins shows that they belong to the IGT (LAZY1/DRO1/TAC1) protein family [9,12,13]. The members of this family do not share a high sequence homology; they are characterized by five short, conserved motifs called domains I–V, not all of which appear in all members. The family name is based on an isoleucine–glycine–threonine motif at the N-terminus of domain II that is essential for directional gravity information [9,12,13]. Seven IGT proteins are known in Arabidopsis and six in rice [8,9,13]. The Arabidopsis proteins LAZY1 and LAZY5, as well as a rice protein, *Os*LAZY1, belong to the LAZY1 clade. The DRO1 clade contains three proteins from Arabidopsis (DRO1, DRO2, and DRO3) [9] and four proteins from rice (DRO1, DRL1, DRL2, and DRL3) [7,8]. The TAC1 clade contains only one protein, TAC1, in Arabidopsis and in rice [9,13].

Genes encoding TAC1-like proteins were identified based on their effects on the regulation of the gravitropic responses of leaves or of the lateral branches of shoots, whereas LAZY1-like proteins, like DRO1, where identified based on their effects on the gravitropic response of roots. Meanwhile, both LAZY1- and DRO1-like proteins were found to regulate gravitropic responses in shoots [13].

Regulatory networks involving IGT proteins have been partially characterized in different plant species [14,15,16,17,18]. In wheat, the *Ta*ARF1 protein binds to the promoter of the *TaBNDRO1* gene [14]; similarly, in rice, the *Os*ARF1/*Os*ARF23 protein binds to the promoter of *OsDRO1* [7]. Moreover, the *Ta*TOPLESS protein, known as a cofactor of Aux/IAA proteins [19], interacts with the *Ta*ANDRO1, *Ta*BNDRO1, and *Ta*DNDRO1 proteins. Altogether, currently available data support that the expression of at least *TaBNDRO1* should be auxin-dependent [14], like that of *OsDRO1* and *OsDRL1* [7,8]. In rice, the interaction between the cytoplasmic protein *Os*LAZY1 and the membrane-associated protein *Os*BRXL4 apparently led to a change in *Os*LAZY1 conformation and to its redistribution from the cytoplasm to the nucleus [15]. In Arabidopsis, many interactors were identified [16,17,18]. The expression of *AtDRO1* (*AtLAZY4*) has been shown to be regulated by the transcription factor *At*HY5 in roots [17]. The interaction of IGT proteins *At*LAZY1, *At*DRO2 (*At*LAZY3), and possibly *At*DRO3 (*At*LAZY2) with the REGULATOR OF CHROMOSOME CONDENSATION1 (RCC1)-like domain1 (*At*RLD1) and *At*RLD2 proteins determined the transport of both *At*RLDs from the cytoplasm to the plasmalemma of columella cells, which apparently led to the asymmetric localization of *At*PIN3, the redistribution of auxin fluxes, and a change in the root growth direction [16]. According to the model of Chen et al. (2023), gravistimulation triggers the *At*MKK/*At*MAPK-mediated phosphorylation of *At*LAZY proteins, enhancing the interaction of *At*LAZYs with *At*TOC proteins on the surface of amyloplasts in columella cells. Amyloplast redistribution then causes the redistribution of *At*LAZYs from the amyloplast surface to the new lower side of the plasma membrane in columella cells, resulting in the formation of a new root tip growth vector [18].

Despite attempts to explore the function of *DRO1*-like genes, it remains unclear whether the characterized mechanisms of lateral root angle formation are universal. Morphological data on emerging lateral roots have shown differences between Arabidopsis and several other plant species. In Arabidopsis, the angle between the main and lateral root is formed gradually during lateral root growth [20,21]; therefore, it could be termed the “lateral root growth angle”. For a short period after emergence, lateral roots grow perpendicular to the longitudinal axis of the parental root. The lateral root angle begins to form when a lateral root reaches a length of 1–2 mm, reaching 70°, and gradually continues to decrease to 20–30° at a lateral root length of 3.5–4.5 mm [20,21]. However, in several phylogenetically unrelated plant species, lateral roots are already sloped at a gravitropic setpoint angle of 60–80° before emergence from the parental root. This morphological parameter, termed in this study the “lateral root slope angle”, is typical for *Cucurbita pepo* [22,23], *Cucurbita maxima* [24], and the aquatic fern *Ceratopteris pteridoides* [25], where lateral root initiation takes place within the apical meristem of the parental root. A slope angle was also observed for lateral roots of *Magnolia virginiana*, poplar (*Populus nigra*), and sunflower (*Helianthus annuus*) [24], where lateral root formation is initiated above the elongation zone. However, it remains unclear whether a mechanism involving the action of *DRO1*-like genes is involved in lateral root slope angle formation in the listed plant species.

In this study, the role of homologs of rice and Arabidopsis DRO1 in cucumber (*Cucumis sativus*) was characterized. Cucumber lateral root primordia, like the lateral root primordia of *Cucurbita pepo* or *Cucurbita maxima*, form a slope angle before the newly formed lateral root emerges from the parental root. A phylogenetic analysis of IGT protein family members in cucumber led to the identification of three putative orthologs of DRO1 in rice and Arabidopsis. The influence of exogenous auxin on the expression of *CsDRO1*, *CsDRO1-LIKE1* (*CsDRO1L1*), and *CsDRO1-LIKE2* (*CsDRO1L2*) was analyzed. Only one gene, *CsDRO1L1*, was shown to be auxin-responsive. An analysis of promoter–reporter fusions showed that the *CsDRO1*, *CsDRO1L1*, and *CsDRO1L2* genes were expressed in the root meristem in cell files of the central cylinder, endodermis, and cortex; all three genes demonstrated different expression patterns in cucumber roots with only partial overlap.

Knockout of individual *CsDRO1*, *CsDRO1L1*, and *CsDRO1L2* genes was performed in transgenic hairy roots via the CRISPR/Cas9-editing system. These knockouts of individual genes did not affect the formation of the slope angle during lateral root primordia development in the cucumber parental root.

## 2. Results

### 2.1. Identification of the Putative Ortholog(s) of Arabidopsis and Rice DEEPER ROOTING1 (DRO1) in Cucumis sativus by Phylogenetic Analysis

**Figure 1:**
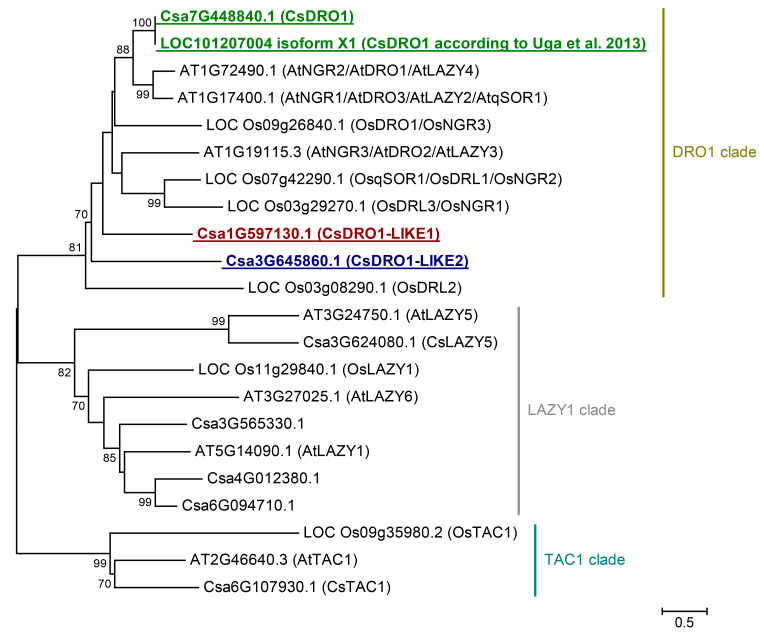
Phylogenetic tree of IGT (LAZY1/DRO1/TAC1) proteins from *Arabidopsis thaliana*, *Oryza sativa,* and *Cucumis sativus*. Putative *C. sativus* ortholog of *At*DRO1 and *Os*DRO1 as well as two DRO1-LIKE cucumber proteins are underlined and labeled in bold in green, red, and blue, respectively. A reference is included for the *C. sativus* DRO1 protein identified previously [7]. The phylogenetic tree was constructed based on a Clustal Omega alignment. Evolutionary analyses were carried out in MEGA7 software using the maximum likelihood method with 1000 bootstrap replicates based on the gamma distributed (+G) Jones–Taylor–Thornton model with invariant sites (+I). Gene ID prefixes: AT, *A. thaliana* based on the Arabidopsis Information Resource; Csa, *C. sativus* cv Chinese Long v. 2 based on Cucurbit Genomics Database v1; LOC101207004 isoform X1, *C. sativus* based on NCBI; LOC_Os, *O*. *sativa* v7.0 based on Phytozome v13. Scale bar: 0.5 amino acid substitutions per site.

Seven Arabidopsis and six rice proteins of the IGT (LAZY1/DRO1/TAC1) family were used for searches in the cucumber proteome. Eight IGT proteins were identified (Figure 1). A phylogenetic analysis of IGT proteins from cucumber showed that, like in Arabidopsis and rice [9,13], they can be divided into three major clades: DRO1, LAZY1, and TAC1. The DRO1 clade contains three cucumber proteins (gene IDs from Cucurbit Genomics Database v1: Csa1G597130.1; Csa3G645860.1; Csa7G448840.1 (=LOC101207004 isoform X1; gene ID from NCBI)). Four cucumber proteins (gene IDs: Csa3G565330.1; Csa3G624080.1; Csa4G012380.1; Csa6G094710.1) belong to the LAZY1 clade. The TAC1 clade consists of a single cucumber protein, Csa6G107930.1.

The phylogenetic analysis revealed three DRO1-like proteins in the cucumber proteome, named *Cs*DRO1, *Cs*DRO1-LIKE1 (*Cs*DRO1L1), and *Cs*DRO1-LIKE2 (*Cs*DRO1L2) (gene IDs: Csa7G448840.1, Csa1G597130.1, and Csa3G645860.1, respectively), based on their amino acid similarity and identity with DRO1 proteins of Arabidopsis and rice (Figure 1, Table 1 and Table 2). The *Cs*DRO1 protein showed a 63.5% amino acid similarity and a 51.5% identity with *At*DRO1, and a 51.7% similarity and 37.2% identity with *Os*DRO1 (Table 1 and Table 2). At the same time, *Cs*DRO1 displayed a high amino acid similarity with other proteins, *At*DRO3 and *Os*DRL1: respectively, 64.5% amino acid similarity and 52% identity with *At*DRO3, and 48.2% similarity and 36.2% identity with *Os*DRL1 (Table 1 and Table 2).

The phylogenetic analysis did not unambiguously determine whether *Cs*DRO1 is the putative ortholog of *At*DRO1/*Os*DRO1. Yet, as *Cs*DRO1 shared less similarity with *At*DRO2, *Os*DRL2, and *Os*DRL3, it is unlikely to represent an ortholog of any of these three proteins (Table 1 and Table 2). The *Cs*DRO1L2 protein shared the lowest similarity with both Arabidopsis and rice DRO proteins (Table 1 and Table 2). *Cs*DRO1L2 showed 25.4–27% amino acid similarity and 15–17% identity with all known Arabidopsis DRO proteins (Table 1). The amino acid similarity and identity of *Cs*DRO1L2 with rice DRO proteins was also very low, 20.3–24.3% and 9.3–13%, respectively (Table 2). The *Cs*DRO1L1 protein had an intermediate position on the phylogenetic tree, between *Cs*DRO1 and *Cs*DRO1L2, in relation to Arabidopsis and rice DRO proteins (Figure 1, Table 1 and Table 2). Like *Cs*DRO1, *Cs*DRO1L1 shared a branch with pairs of Arabidopsis and rice proteins, *At*DRO1/*At*DRO3 and *Os*DRO1/*Os*DRL1 (Table 1 and Table 2). Based on sequence similarity, *Cs*DRO1L1 is not an ortholog of *At*DRO2, *Os*DRL2, and *Os*DRL3 (Table 1 and Table 2).

Apart from *Cs*DRO1 and *Cs*DRO1Ls, we also identified and named several cucumber proteins that were sister-paired with Arabidopsis and rice IGTs: *Cs*LAZY5 (gene ID: Csa3G624080.1), *Cs*TAC1 (gene ID: Csa6G107930.1), and three *Cs*LAZY1-like proteins (gene IDs: Csa3G565330.1; Csa4G012380.1; Csa6G094710.1) (Figure 1). We also found that a protein recently reported as *At*NGR1/*At*DRO3/*At*LAZY2 (gene ID: AT1G17400.1) in the Arabidopsis Information Resource is identical to a protein previously identified as *At*qSOR (Figure S1) [8]; all names for this protein are denoted in the phylogenetic tree (*At*NGR1/*At*DRO3/*At*LAZY2/*At*qSOR) (Figure 1).

### 2.2. Expression Levels of Cucumber DRO Genes Differ between Organs and in Their Response to Exogenous Auxin

**Figure 2:**
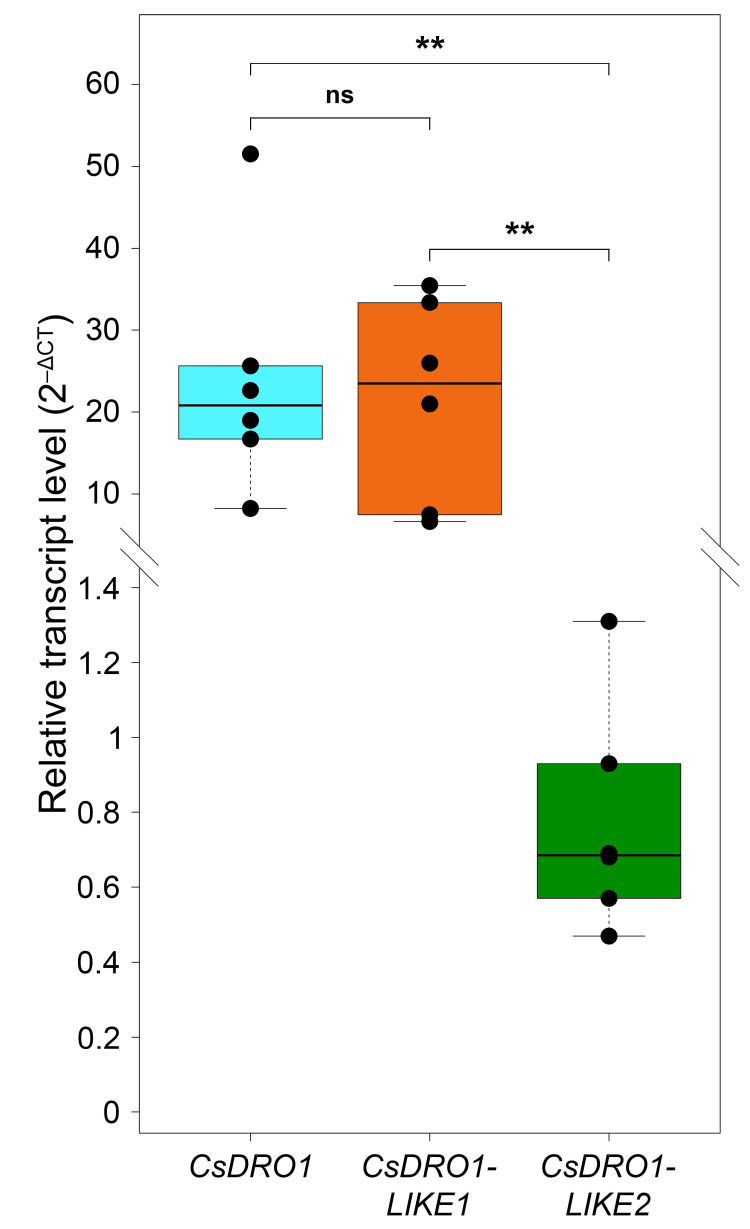
Expression of *Cucumis sativus DRO* genes in roots. RT-qPCR analysis was performed using RNA isolated from the first centimeter of the primary root of seven-day-old seedlings. Statistical analysis using an unpaired two-sample Wilcoxon test showed significant differences (**, *p* < 0.01) between *CsDRO1-LIKE2* and *CsDRO1* or *CsDRO1-LIKE1*, respectively, whereas no significant differences were observed between *CsDRO1* and *CsDRO1-LIKE1* expression levels (ns). The *y* axis indicates the relative transcript level (2^–ΔCT^ method).

It was reported previously that not all Arabidopsis *DRO1*-like genes were expressed in roots [9]. Accordingly, RT-qPCR analysis was performed to confirm whether all cucumber *DRO* genes identified in this study (*CsDRO1*, *CsDRO1-LIKE1* (*CsDRO1L1*), and *CsDRO1-LIKE2* (*CsDRO1L2*)) were expressed in roots (Figure 2). The expression levels of *CsDRO1L2* in the roots were significantly lower than those of *CsDRO1* or *CsDRO1L1*. The transcript levels of *CsDRO1L2* in the roots were 12–74-fold lower than those of *CsDRO1* and 9–75-fold lower than those of *CsDRO1L1*, whereas no significant differences were detected between the expression levels of *CsDRO1* and *CsDRO1L1* (Figure 2).

**Figure 3:**
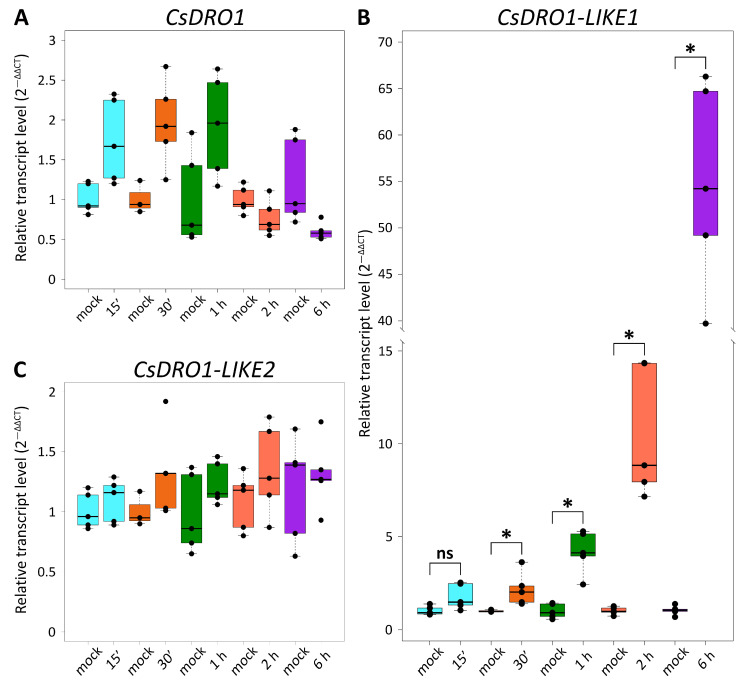
Expression of *Cucumis sativus DRO* genes in response to treatment with 10 µM of NAA. (**A**) *CsDRO1*; (**B**) *CsDRO1-LIKE1*; (**C**) *CsDRO1-LIKE2*. Four-day-old *C. sativus* seedlings were incubated with NAA for 15 min (15′), 30 min (30′), 1 h, 2 h, and 6 h, respectively. Statistical analysis using Wilcoxon test showed significant differences (*, *p* < 0.05) between *CsDRO1-LIKE1* expression in mock-treated vs. NAA-treated roots at all time points, except for the 15 min time point where no significant differences were observed (ns). RT-qPCR analysis was performed using RNA isolated from the first distal centimeter of the primary root. The *y* axis indicates the relative transcript level (2^–ΔΔCT^ method).

To assess whether *CsDRO1*, *CsDRO1L1*, or *CsDRO1L2* are closely related orthologs or paralogs of *OsDRO1* or *OsDRL1*, the expression levels of *CsDRO1*, *CsDRO1L1*, and *CsDRO1L2* were analyzed in cucumber roots in response to exogenous auxin (Figure 3). RT-qPCR analysis showed that the transcript levels of *CsDRO1* and *CsDRO1L2* did not change in response to treatment with 10 µM of naphthaleneacetic acid (NAA) for 15 min, 30 min, 1 h, 2 h, or 6 h (Figure 3A,C). While the transcript levels of *CsDRO1L1* were not altered in response to treatment with 10 µM of NAA for 15 min, a positive response to auxin was demonstrated at all later time points. The transcript levels were increased 2–3-fold after 30 min; 3–7-fold after 1 h of treatment; 7–15-fold after 2 h of treatment; and 40–66-fold after 6 h.

### 2.3. Expression Patterns of Cucumis sativus DRO Genes in the Root Tip and during Lateral Root Emergence Based on Promoter–Reporter Fusions in Transgenic Hairy Roots

**Figure 4:**
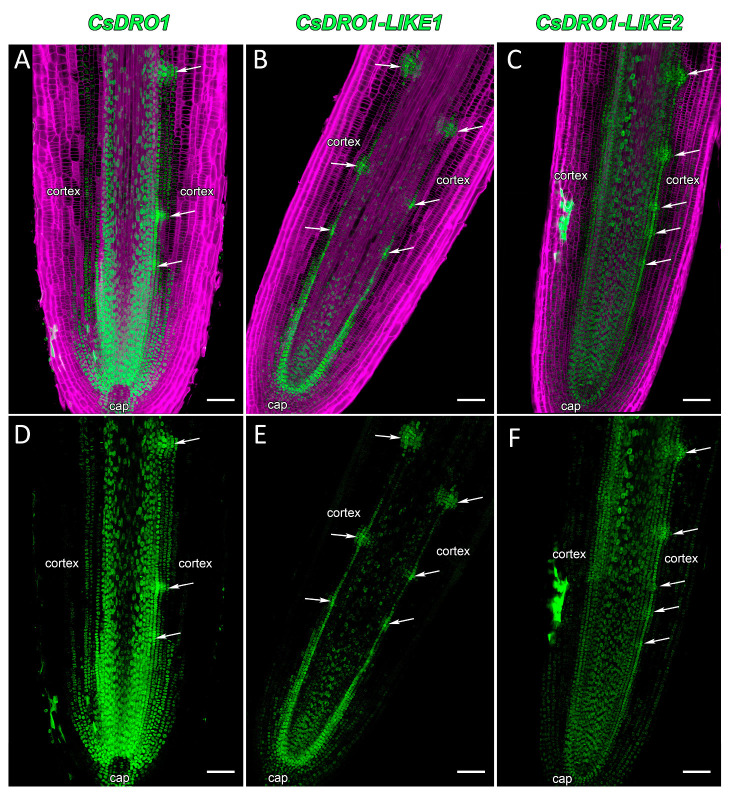
Localization of *CsDRO1*, *CsDRO1-LIKE1*, and *CsDRO1-LIKE2* expression in *Cucumis sativus* root tips (*pCsDRO1::mNeonGreen-H2B*, *pCsDRO1L1::mNeonGreen-H2B*, *pCsDRO1L2::mNeonGreen-H2B)*. Confocal laser scanning microscopy of longitudinal vibratome sections. Expression of *CsDRO1* (**A**,**D**) and *CsDRO1L2* (**C**,**F**) was maintained in the central cylinder, cortex, and developing lateral root primordia (white arrows). (**B**,**E**) Expression of *CsDRO1L1* was maintained in the central cylinder, endodermis, and lateral root primordia (white arrows). Green channel, fluorescence of mNeonGreen; magenta channel, SR2200-stained cell walls. Single optical sections. Scale bars, 100 µm.

Despite the fact that the *Cs*DRO1 and *CsDRO1L*2 proteins are phylogenetically distant from each other (Figure 1), the expression patterns of the corresponding genes were similar in cucumber root tips (Figure 4A,C,D,F). Both promoters, *pCsDRO1* and *pCsDRO1L2*, were active in the root meristem, in the central cylinder, and in the primary cortex cells, starting from the initial cells (Figure 4A,C,D,F). The expression of both genes in the primary cortex cell files was attenuated with increasing distance from the initial cells (Figure 4D,F). Notably, *pCsDRO1* was also active in protophloem cells, and was not active in the quiescent centre (Figure 4A,D). Expression maxima (the strongest reporter signal in nuclei) of *CsDRO1* and *CsDRO1L2* were localized in pericycle cells (Figure 4A,C,D,F). The maximum of *CsDRO1L1* expression was detected in the endodermal cell file, starting from the initial cells and gradually weakening (Figure 4B,E). *pCsDRO1L1* was also active in the inner cortical layers and the apical part of the central cylinder, but its activity gradually faded with increasing distance from the initial cells, and completely disappeared at a distance of approximately 850 μm (Figure 4B,E). The *CsDRO1L1* expression maximum was maintained in the endodermal cells involved in the formation of lateral root primordia. *pCsDRO1*, *pCsDRO1L1*, and *pCsDRO1L2* were active in the root cap, but the activity of *pCsDRO1L2* was much lower in this area (Figure 4F). *pCsDRO1* and *pCsDRO1L2*, but not *pCsDRO1L1,* were active in the root cap columella (Figure S2). As already shown by RT-qPCR, the overall level of *CsDRO1L2* expression was significantly lower than that of *CsDRO1* or *CsDRO1L1* (Figure 4D–F and Figure S2D–F).

**Figure 5:**
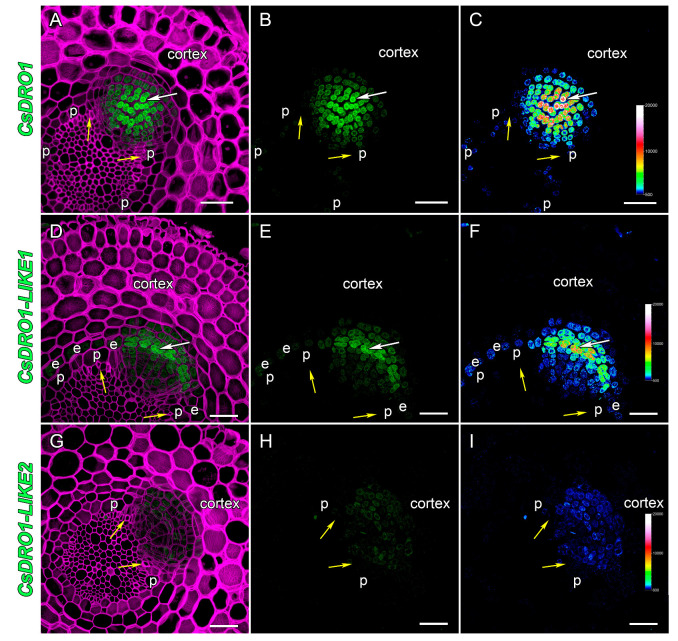
Localization of *DRO1*, *CsDRO1-LIKE1*, and *CsDRO1-LIKE2* expression in lateral root primordia of *Cucumis sativus* roots (*pCsDRO1::mNeonGreen-H2B*, *pCsDRO1L1::mNeonGreen-H2B*, *pCsDRO1L2::mNeonGreen-H2B)*. Confocal laser scanning microscopy of vibratome cross sections at a distance of about 800 μm from the initial cells. (**A**–**C**) Expression maxima of *CsDRO1* were maintained in the central part of a lateral root primordium (white arrows), while lower expression levels were found in the pericycle. (**D**–**F**) Expression maxima of *CsDRO1L1* were maintained in the endodermal part of developing lateral root primordia (white arrows) and the endodermis or the parental root. (**G**–**I**) Expression of *CsDRO1L2* was reduced in the lateral root primordia (white arrows), and absent in the central cylinder. Green channel, fluorescence of mNeonGreen; magenta channel, SR2200-stained cell walls. Maximum intensity projection of z-series: (**A**–**C**) of 9 optical sections, 7 µm in depth; (**D**–**F**) of 30 optical sections, 23 µm in depth; and (**G**–**I**) of 28 optical sections, 21 µm in depth. The heatmap shows color-coded fluorescence signal intensities for the green channel; the quantification scale is the same for all images. White arrows—promoter activity maxima; yellow arrows—phloem poles; e, endodermis; p, pericycle. Scale bars, 50 µm.

During the development of lateral root primordia in the elongation zone (Figure 4 and Figure 5), the maxima of expression of *CsDRO1* and *CsDRO1L1* were distributed differently. The activity of *pCsDRO1* was maintained in the central part of the developing lateral root primordia (Figure 5A–C), and the expression levels of *CsDRO1* remained high (Figure 5C). The expression maxima of *CsDRO1L1* were maintained in the part of developing lateral root primordia that is of endodermal origin (Figure 5D–F). The intensity of the reporter signal of *CsDRO1L1* was approximately half that of *CsDRO1* (Figure 5C,F). The expression levels of *CsDRO1L2* were significantly (approximately 20-fold) lower in developing primordia compared to those of the other two genes (Figure 5G,I).

**Figure 6:**
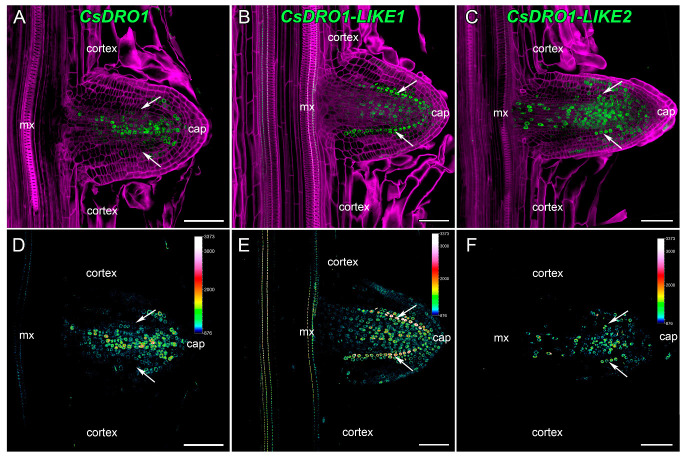
Localization of *DRO1*, *CsDRO1-LIKE1*, and *CsDRO1-LIKE2* expression in emerging lateral roots of *Cucumis sativus* (*pCsDRO1::mNeonGreen-H2B*, *pCsDRO1L1::mNeonGreen-H2B*, *pCsDRO1L2::mNeonGreen-H2B)*. Confocal laser scanning microscopy of longitudinal vibratome sections. (**A**,**D**) *CsDRO1* promoter activity maxima in the central cylinder. (**B**,**E**) *CsDRO1L1* promoter activity maxima in the endodermis. (**C**,**F**) The *CsDRO1L2* promoter activity maximum in the central cylinder is lower than that of *CsDRO1*. Green channel, fluorescence of mNeonGreen; magenta channel, SR2200-stained cell walls. The heatmap shows color-coded fluorescence signal intensities for the green channel; the quantification scale is the same for all images. White arrows—endodermis; mx, metaxylem. Single optical sections. Scale bars,100 µm.

The expression patterns of *CsDRO1*, *CsDRO1L1*, and *CsDRO1L2* in the emerging lateral roots were very similar to the patterns detected in the parental root meristem (Figure 4 and Figure 6). Expression maxima were present mainly in the central cylinder, and in some cortex cells, in the apical part of the meristem (Figure 6). In the root cap, only *CsDRO1L1* expression was detected at this development stage (Figure 6B,E). It is important to note that the *CsDRO1L1* expression maxima were maintained in the endodermis, as was already shown for the parental root meristem (Figure 4 and Figure 5). The expression level of *CsDRO1L2* was clearly lower than those of *CsDRO1* or *CsDRO1L1* (Figure 6C,F).

### 2.4. CRISPR/Cas9-Mediated Genome Editing in Transgenic Cucumber Roots

**Figure 7:**
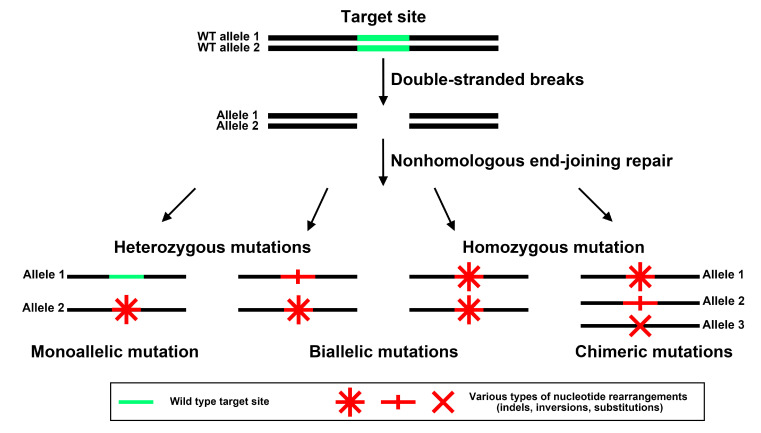
Variants of different mutations caused by the CRISPR/Cas9-editing system.

Cucumber is a diploid plant species (2n = 2x = 14) [26]. Since cucumber genes have one allele in each sister chromosome, the CRISPR/Cas9 system can induce four variants of mutations in alleles of the target gene (Figure 7). If one of the wild-type alleles remains intact and the other one carries a mutation, the mutations are called monoallelic. This type of mutation cannot result in a complete loss of function, since a wild-type allele of the gene is retained. If two alleles are mutated at the same time, the mutations are called biallelic. There are two subgroups of biallelic mutation events. If both alleles carry different mutations, they are biallelic heterozygous mutations. If both alleles carry the same mutation, the resulting plant is biallelic homozygous. Different mutations in one or both alleles of the same gene are called chimeric. Hetero- and homozygous biallelic mutations are the most important ones for the functional analysis of genes, as they may lead to the complete disruption of gene function (Figure 7).

#### 2.4.1. Identification of Mutations in a Single Cucumber DRO1, DRO1L1 or DRO1L2 Gene

A screening of CRISPR/Cas9-mediated *CsDRO1* genome editing events was performed on 66 transgenic cucumber roots. Among them, biallelic homozygous mutations were found in seven roots (Figure S3), monoallelic mutations in two roots, while 27 roots harbored chimeric mutations in the alleles of the *CsDRO1* gene. No mutations were detected in three roots. Twenty seven roots were excluded from further analysis because of the unclear character of the mutations they carried (overlapping peaks on the sequence chromatograms’ start in the middle of the target site).

Ninety three roots were selected for a further analysis of mutation events caused by the CRISPR/Cas9 system in *CsDRO1L1*. Three roots carried biallelic heterozygous mutations (Figure S4), twelve roots carried biallelic homozygous mutations (Figure S5), seven roots carried monoallelic mutations, and 52 roots carried chimeric mutations. Ten roots had no mutations in the *CsDRO1L1* sequence. Nine roots were excluded from further analysis for the same reason as the 27 roots with mutated *CsDRO1* sequences mentioned above: overlapping peaks.

Eighty nine roots were analyzed for genome editing events in the *CsDRO1L2* sequence. Several edited *CsDRO1L2* sequences were detected: six roots carried biallelic heterozygous mutations (Figure S6), twelve roots carried biallelic homozygous mutations (Figure S7), one root carried a monoallelic mutation, and 21 roots carried chimeric mutations. In nine roots, no *CsDRO1L2* gene mutations were detected. The genomic DNA of 40 roots yielded PCR products with overlapping chromatogram sequence peaks in the middle of the target site, so these roots were not analyzed further.

Thus, the overall efficiency of gene editing was 95.45% (*n =* 66) for *CsDRO1*, 89.25% (*n =* 93) for *CsDRO1L1*, and 89.89% (*n =* 89) for *CsDRO1L2*.

#### 2.4.2. Knockout of Any Individual DRO Gene Does Not Change the Lateral Root Slope Angle

Experiments were carried out on single transgenic roots without the regeneration of transgenic plants. The analysis of the lateral root slope angle in the transgenic roots of the control group and the groups with characterized mutations of *CsDRO1*, *CsDRO1L1,* or *CsDRO1L2*, obtained via gene editing, was carried out only for those roots in which the nature of the mutations met two obligatory conditions. First, the identified mutations had to be biallelic heterozygous or biallelic homozygous (Figures S3–S7). Second, the detected mutations had to be frame-shift mutations leading to the occurrence of premature stop codons. An analysis of the encoded amino acid sequence was performed in silico using the ExPASy translate tool (web.expasy.org/translate, accessed on 13 December 2023) [27]. Roots harboring deletions leading to non-frame-shift mutations were excluded from further morphological analysis since it would have been difficult to determine whether the loss of several amino acids led to a significant disturbance of the function of the encoded protein.

**Figure 8:**
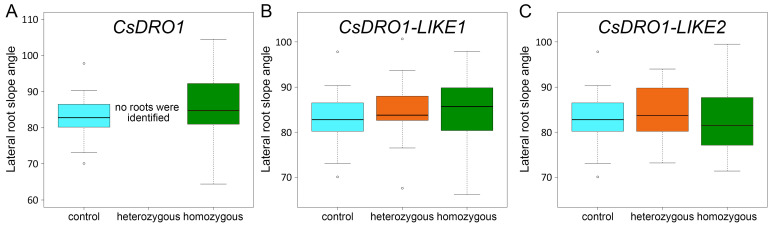
Values of lateral root slope angle relative to the vertical axis of the parental root in transgenic roots of cucumber (*Cucumis sativus*). Control group: roots transformed with the empty pKSEe401R vector for gene editing (*n =* 11: number of transgenic roots; *n =* 33: number of lateral roots). (**A**) Roots with confirmed mutations of *CsDRO1* via gene editing: biallelic homozygous mutations (*n =* 7: transgenic roots; *n =* 28: lateral roots). (**B**) Roots with confirmed mutations of *CsDRO1-LIKE1* via gene editing: biallelic heterozygous mutations (*n =* 3: transgenic roots; *n =* 13: lateral roots); biallelic homozygous mutations (*n =* 12: transgenic roots; *n =* 56: lateral roots). (**C**) Roots with confirmed mutations of *CsDRO1-LIKE2* via gene editing: biallelic heterozygous mutations (*n =* 5: transgenic roots; *n =* 22: lateral roots); biallelic homozygous mutations (*n =* 8: transgenic roots; *n =* 30: lateral roots). Statistical analysis using Wilcoxon test showed no significant differences (*p* < 0.05).

Transgenic roots, transformed with an empty editing vector, pKSEe401R, were used as the control group. In 11 roots of the control group, the slope angle of 33 lateral roots was measured, resulting in an average value of 82.5 ± 1.02° (Figure 8). For seven roots carrying biallelic homozygous mutations in the *CsDRO1* gene, the slope angle was 85.49 ± 1.73° (average of 28 lateral roots) (Figure 8A).

Fifteen roots carrying biallelic mutations (hetero- or homozygous) in the *CsDRO1L1* gene were selected. The average of lateral root slope angle was 84.81 ± 2.21° (*n* = 13) in three roots carrying biallelic heterozygous mutations, and 85.25 ± 0.86° (*n* = 56) in 12 roots with biallelic homozygous mutations (Figure 8B).

Although 18 roots carrying biallelic hetero- or homozygous mutations in the *CsDRO1L2* gene were identified, only 13 roots were selected for further analysis. In five roots with biallelic mutations in the *CsDRO1L2* gene (one root with heterozygous mutation and four with homozygous mutations), deletions were detected that did not lead to frameshifts. Thus, further analyses were performed only for five heterozygous roots (22 lateral roots) and for eight homozygous roots (30 lateral roots). The average slope angles were 84.61 ± 1.26° and 82.05 ± 1.29°, respectively (Figure 8C).

In summary, no statistically significant changes in the lateral root slope angle relative to the vertical axis of the parental root were revealed for roots with confirmed mutations of *CsDRO1*, *CsDRO1L1,* or *CsDRO1L2*.

## 3. Discussion

We identified eight IGT family proteins (LAZY1/DRO1/TAC1) in cucumber, which is one more protein than in Arabidopsis [9,10] and two more than in rice [8,10]. Thus, a possible duplication of one or two cucumber genes encoding IGT protein(s) can be assumed. Despite the unequal number of proteins between the compared plant species, the IGT proteins in cucumber, as well as in Arabidopsis and rice [9,13], showed a clear separation into three major clades: DRO1, LAZY1, and TAC1. However, not all clades had equal numbers of proteins in the different plant species, except that only one protein was identified in the TAC1 clade of each species (*Os*TAC1-*At*TAC1-*Cs*TAC1 branch). The numbers of proteins in the LAZY1 clade varied. For example, the phylogenetic pair *At*LAZY5-*Cs*LAZY5 was clearly distinguishable, while no rice ortholog of these proteins was found. The *At*LAZY6 protein had no putative orthologs in either cucumber or rice. Both rice and Arabidopsis had one LAZY1 protein, whereas three LAZY1-like proteins were identified in cucumber. The DRO1 clade also showed unequal numbers of proteins, with three proteins in Arabidopsis and four in rice. Only one (gene ID: LOC101207004) [7] or two (gene IDs: Csa_1G597130, Csa_7G448840) [8] DRO1 proteins had been described in cucumber. We showed the presence of three DRO1-like proteins in cucumber by phylogenetic analysis: *Cs*DRO1 (gene ID: Csa7G448840.1), *Cs*DRO1-LIKE1 (gene ID: Csa1G597130.1), and *Cs*DRO1-LIKE2 (gene ID: Csa3G645860.1) (Figure 1). The *Cs*DRO1L2 protein is clearly more distantly related to all known rice or Arabidopsis DRO proteins than *Cs*DRO1 and *Cs*DRO1L1. The *Cs*DRO1 and *Cs*DRO1L1 proteins cannot be phylogenetically separated from the *At*DRO1-*At*DRO3 pair due to equal amino acid similarity to both Arabidopsis proteins (Table 1). Similarly, *Cs*DRO1 and *Cs*DRO1L1 proteins show equal levels of similarity with *Os*DRO1 and *Os*DRL1 (Table 2).

The analysis of transcription levels of all cucumber *DRO* genes, *CsDRO1*, *CsDRO1L1*, and *CsDRO1L2*, in roots showed that all three genes were expressed in roots (Figure 2). While both *CsDRO1* and *CsDRO1L1* had high sequence similarity not only with *AtDRO1*, but also with *AtDRO3* (Figure 1, Table 1), it should be noted that according to semi-quantitative PCR data, *AtDRO1* transcripts were found in roots, while *AtDRO3* was not expressed there [9], a result that shed doubt on the assumption of orthology. However, Yoshihara and Spalding [10] showed, using promoter–GUS fusions, that *AtDRO3* (*AtLAZY2*) as well as *AtDRO1* (*AtLAZY4*) and *AtDRO2* (*AtLAZY3*) promoters were active in roots.

We found strong differences between the expression levels of *CsDRO1L2* and *CsDRO1*/*CsDRO1L1* in cucumber roots: *CsDRO1L2* was expressed at significantly lower levels than *CsDRO1* and *CsDRO1L1* (Figure 2). These data were supported by a bioinformatic analysis of data from two cucumber root transcriptomes (BioProject IDs: PRJNA271595; PRJNA80169) [28,29] available in the Cucurbit Expression Atlas in the Cucurbit Genomics Database v1: the RPKM values for *CsDRO1L2* were 2–5-fold lower than for *CsDRO1* and *CsDRO1L1*. Taken together with the cell-specific expression patterns, these data might suggest a different role of *CsDRO1L2* in cucumber root development.

Auxin is a key factor for not only the initiation and development of lateral roots [23,30], but also for the lateral root growth angle [31]. The lateral roots of *pin3pin4*, *pin3pin7*, and *pin3pin4pin7* mutants with impaired polar auxin transport showed a reduced ability to bend in the direction of the gravity vector, thus forming a radial root system with lateral root angles close to 90°. It is possible that the asymmetric distribution of auxin by transporters *At*PIN3/*At*PIN4/*At*PIN7 leads to an accumulation of auxin on one side of a lateral root. The difference in the rate of cell elongation on different sides of the lateral root at the stage after emergence determines its response to gravity and leads to the formation of an axial root system with lateral root angles lower than 90° [31].

The *OsDRO1* gene is considered to be one of targets expressed in response to uneven auxin distribution, mediating the formation of the adventitious root growth angle [7]. *OsDRO1* and *OsDRO1L1* have been shown to be early auxin response genes: 30 min after treatment of roots with 10 μM of 2,4-dichlorophenoxyacetic acid, *OsDRO1* and *OsDRO1L1* (*OsqSOR1*) expression levels decreased [7,8]. Uga et al. [7] concluded that the inhibitory effect of auxin on *OsDRO1* expression caused the asymmetric growth of cells of the root elongation zone, thereby leading to a certain angle of penetration of the rice root system into the soil.

An analysis of changes in the expression levels of *CsDRO1*, *CsDRO1L1*, and *CsDRO1L2* genes in response to treatment with naphthaleneacetic acid (NAA) revealed that only *CsDRO1L1* is an early auxin response gene (Figure 3B). However, in contrast with *OsDRO1* [7], *CsDRO1* expression levels were not significantly affected by NAA (Figure 3A); instead, the expression of *CsDRO1L1* was auxin-responsive. However, this response was positive, instead of negative as for *OsDRO1:* the expression of *CsDRO1L1* was significantly up-regulated in response to NAA (Figure 3B). The expression levels of *CsDRO1L2*, like those of *CsDRO1*, did not change significantly in response to auxin (Figure 3A,C). Thus, the data on the auxin responsiveness of *DRO1*-like cucumber genes do not answer the question as to whether *CsDRO1* and/or *CsDRO1L1* represent orthologs of *OsDRO1* and/or *OsDRL1*.

A comparison of the auxin sensitivity of cucumber *DRO1*-like genes with that of the corresponding Arabidopsis genes was equally inconclusive. At least two of the three Arabidopsis *DRO* genes, *AtDRO1* and *AtDRO2*, are most likely not regulated by auxin [32,33,34]. We performed a search for *AtDRO1*, *AtDRO2*, and *AtDRO3* (gene IDs: AT1G72490, AT1G19115, and AT1G17400, respectively) in the dataset of Stigliani et al. [33], compiled from Arabidopsis gene expression profiling data in response to auxin treatment. The IDs of all three genes were absent both from the group of early-activated genes and from the group of early-repressed genes, indicating that Arabidopsis *DRO* genes are not early auxin-responsive genes [33]. The analysis of the Arabidopsis seedling transcriptome after a treatment with 10 μM of indole-3-acetic acid (IAA) for 6 h showed that the IDs of two of the three *DRO* genes, *AtDRO1* and *AtDRO2*, fall into the group of DEGs non-regulated by auxin [32]; it should be taken into account, however, that Omelyanchuk et al. [32] did not analyze the early response. To date, transcriptomic data on the auxin regulation of Arabidopsis *DRO* gene expression have been confirmed by RT-qPCR only for the *AtDRO1* gene. *AtDRO1* expression did not change in response to a treatment of Arabidopsis seedlings with 1 or 10 μM of IAA for 30 min, 1 h, and 6 h [34], similar to the expression of *CsDRO1* and *CsDRO1L2* in cucumber. Altogether, the data on the effects of auxin on the expression of *DRO1*-like genes in Arabidopsis, rice, and cucumber suggest that transcriptional regulation by auxin might not be obligatory for the function of some *DRO1*-like genes in the establishment of the root gravitropic response.

The expression domains of *CsDRO1*, *CsDRO1L1*, and *CsDRO1L2* genes were quite different in comparison with the expression patterns described for *AtDRO1*, *AtDRO2*, *AtDRO3*, *OsDRO1*, and *OsDRL1*/*OsqSOR1*. For example, the expression of *AtDRO1* (*AtNGR2*), *AtDRO2* (*AtNGR3*), and *AtDRO3* (*AtNGR1*) was restricted to root cap cells [35]. These data were supported by GUS staining results for *AtDRO2* (*AtLAZY3*) and *AtDRO3* (*AtLAZY2*) genes [10]. However, the promoter of the *AtDRO1* gene was active not only in the root cap cells but also in the root vascular cylinder [10], and according to Guseman et al. [9], also in the root cortex at a rather long distance (several centimeters) from the initial cells. In our study, the expression of *CsDRO1*, *CsDRO1L1*, and *CsDRO1L2* was mainly localized in the meristem, in the vascular cylinder, and, to a lesser extent, in the cortex (Figure 4). Promoter activity of *CsDRO1* and *CsDRO1L2* was also detected in the columella cells of the root cap (Figure S2), but was neither especially strong in nor restricted to this zone, in contrast to what was shown for the promoters of *AtDRO2* and *AtDRO3*. The expression of the *OsDRL1*/*OsqSOR1* gene, as of *AtDRO2* and *AtDRO3*, was only detected in root cap cells [8]. However, the transcription of *OsDRO1* took place in the root vascular cylinder and cortex; while an attenuation of *OsDRO1* expression in the cortex was observed with increasing distance from the initial cells, this expression was maintained over centimeters [7].

In short, based on the expression pattern analysis, *CsDRO1*, *CsDRO1L1*, and *CsDRO1L2* most strongly resemble the *AtDRO1* and *OsDRO1* genes, not the root cap-specific *AtDRO2*, *AtDRO3,* or *OsDRL1*/*OsqSOR1* genes. The expression domains of *CsDRO1*, *CsDRO1L1*, and *CsDRO1L2* overlapped in the cell files of the vascular cylinder (Figure 4), which might indicate overlapping functions despite their phylogenetic differences (Figure 1, Table 1 and Table 2) and different responses to auxin (Figure 3). Interestingly, *CsDRO1* expression was maintained in lateral root primordia from initiation to lateral root emergence, and the expression maximum was localized in the pericycle (Figure 4, Figure 5 and Figure 6). At the same time, the expression maximum of *CsDRO1L1* was located in the endodermis of the parental root and in lateral root primordia in cells of endodermal origin (Figure 5). Particularly noteworthy was the finding that the expression levels of *CsDRO1L2*, both according to RT-PCR data and according to promoter activity localization, were significantly lower than those of *CsDRO1* and *CsDROL1* (Figure 5 and Figure 6). This might suggest a different function for *CsDRO1L2*, while the functions of *CsDRO1* and *CsDRO1L1* might overlap.

To elucidate the roles of *CsDRO1*, *CsDRO1L1*, or *CsDRO1L1* in the control of lateral root slope angle formation, they were knocked out individually via CRISPR/Cas9 editing. We analyzed the types of mutations in individual transgenic roots. Mutations resulting from CRISPR/Cas9 editing were categorized as heterozygous, homozygous, biallelic, or chimeric [36,37,38]. We suggest that this classification needs to be clarified further. If we characterize mutations only by the term “heterozygous”, it remains unclear how many mutated alleles ensure heterozygosity: the term can refer to a combination of a wild-type allele and a mutated allele, or to two alleles carrying different mutations. The use of the term “biallelic mutations” unambiguously indicates the number of mutated alleles, but does not give any information about the nature of the mutations which might be homozygous or heterozygous. Thus, in this paper, we propose to expand the existing classification of mutations and to distinguish between monoallelic, biallelic heterozygous, biallelic homozygous, and chimeric mutations (Figure 7).

The editing of *CsDRO1*, *CsDRO1L1*, or *CsDRO1L2* genes performed in this study was highly efficient. We attribute the high percentage of edited roots to the presence of the translational enhancer *OsMac3* [39,40,41,42] upstream of the *Cas9* sequence in the pKSEe401R vector developed for this study. Since the vector was targeting three different sites in one gene simultaneously (Figures S3–S8), we assume that the determining factor for effective editing is the amount of Cas9 protein able to bind to the reading frame targeted by a guide RNA.

Transgenic roots carrying biallelic hetero- or homozygous mutations in either *CsDRO1*, *CsDRO1L1,* or *CsDRO1L2* showed statistically insignificant changes in lateral root slope angles, which suggests that the knockout of only one *DRO1* homolog in cucumber is not sufficient to obtain a phenotypic effect (Figure 8). According to previous studies, the gravitropic response of rice roots is regulated by *OsDRO1* [7] and its paralog *OsDRL1* [8]; however, the slope angle was not quantified in those studies. Thus, in rice, at least two genes are involved in controlling the gravitropically specified angle in rice root branching. Two more *OsDRO1* paralogs with unknown functions, *OsDRL2* and *OsDRL3*, have been identified but not analyzed [8]. In Arabidopsis, apparently only *AtDRO1* (*AtLAZY4*) and *AtDRO3* (*AtLAZY2*) play a role in the formation of the growth angle of lateral roots [10]. The slope angle of lateral roots was significantly reduced in either *dro1* (*lazy4*) or *dro3* (*lazy2*) single mutants compared to the wild type, but in the double-mutant *dro1dro3* (*lazy4lazy2*), the difference was dramatically increased [10]. These data allow us to speculate that the angle of the lateral roots of cucumber might also be regulated by more than one gene.

## 4. Materials and Methods

### 4.1. Plant Material and Bacterial Strains

Cucumber (*Cucumis sativus*) cv. “Kustovoy” and Japonica rice (*Oryza sativa*) cv. “Flagman” were used in this study. Cucumber seeds were purchased from the local provider, Sortsemovosch (Saint Petersburg, Russian Federation). Rice seeds were purchased from the Rice Research Institute (Krasnodar, Russian Federation).

The *Escherichia coli* strain XL-1 Blue [43] was used for molecular cloning. *Rhizobium rhizogenes* (*Agrobacterium rhizogenes*) strain R1000 [44,45], harboring Ri-plasmid pRiA4b, was used for the genetic transformation of cucumber seedlings.

### 4.2. Phylogeny and Bioinformatics

All known *Arabidopsis thaliana* and rice sequences of IGT proteins [8,9,10] were downloaded from the Arabidopsis Information Resource (TAIR, www.arabidopsis.org, accessed on 13 December 2023) [46] and from Phytozome v. 13 (phytozome-next.jgi.doe.gov, accessed on 13 December 2023) [47], respectively. Arabidopsis and rice IGT amino acid sequences were used as queries to find amino acid sequences of *C. sativus* (cucumber, cv Chinese Long v. 2) [29] in the Cucurbit Genomics Database v1 (CuGenDB v1, cucurbitgenomics.org, accessed on 13 December 2023) [48]. The cucumber DRO1 amino acid sequence, identified by Uga et al. [7], was also downloaded from GenBank (ncbi.nlm.nih.gov/genbank, accessed on 13 December 2023) [49] and used for alignment. The alignment was performed using the online Clustal Omega software (www.ebi.ac.uk/Tools/msa/clustalo/, accessed on 13 December 2023) [50] with the default settings. The phylogenetic tree of Arabidopsis, rice, and cucumber IGT proteins was constructed in the MEGA7.0 software [51] using the maximum likelihood method [52] based on the gamma-distributed (+G parameter) [52] Jones–Taylor–Thornton model [53] with invariant sites (+I parameter) [54]. Phylogeny was tested using the bootstrap method with 1000 replications.

### 4.3. Molecular Cloning, Plasmid Construction, and Plant Transformation

#### 4.3.1. Genetic Constructs for Promoter–Reporter Fusions

Three genetic constructs containing the promoter regions of *CsDRO1*, *CsDRO1L1*, and *CsDRO1L2* fused to the *H2B-mNeonGreen* reporter were developed using multisite Gateway technology (Gateway LR Clonase II plus, Thermo Fisher Scientific, Waltham, MA, USA) as described by Kiryushkin et al. [55]. Briefly, the pKGW-RR-MGW binary vector [56], carrying the *pAtUBQ10::DsRED1* screening cassette [57] in the backbone, was used as destination vector. The *mNeonGreen-H2B*-pUC18-entry8 entry vector was used as a donor of the reporter sequence *mNeonGreen-H2B*, and pENTRattR2attL3-*TermAct* was used as a donor of the *AtActin2* gene terminator *TermAct* [55].

All promoter-containing entry vectors were developed based on pENTRattL4attR1_BSAI (Wageningen University, Wageningen, The Netherlands). The upstream sequences containing the putative promoter region and 5′-UTR of the *CsDRO1*, *CsDRO1L1*, and *CsDRO1L2* genes, respectively, were PCR-amplified using genomic DNA of cucumber as template and cloned into pENTRattL4attR1_BSAI using the *Xho*I-*Kpn*I restriction sites (Table 3). The small size of the cloned part of the putative *CsDRO1L1* promoter (808 bp) is explained by the fact that the transcription of this gene is regulated by a so called bidirectional promoter [58,59] of only 837 bp (the second coding region starts in the opposite direction on the minus DNA strand at –838 bp from the ATG of *CsDRO1L1* and belongs to *Csa1G59710.1*).

The LR plus clonase reactions were prepared according to Untergasser [60]. The resulting fusions in all constructs (Figure S8A) were verified by PCR amplification of the fragments and sequencing of the products.

#### 4.3.2. Construction of Vectors for the CRISPR-Cas9-Mediated Genome Editing

The CRISPR/Cas9 vector, pKSEe401R, containing the maize codon-optimized *Cas9* (*zCas9*) enhanced by the 5′-UTR fragment of *OsMac3* [39,40,41,42] and the *pAtUBQ10::DsRED1* screening cassette [57] in the backbone, was constructed as described previously [61]. Briefly, the 158-bp 5′-UTR fragment (from −158 to −1 bp before the ATG) of *OsMac3* (Figure S9) was amplified from Japonica rice genomic DNA followed by cloning into pJET1.2 (CloneJET PCR Cloning Kit, Thermo Fisher Scientific), resulting in pJET1.2-*OsMac3* (Figure S8B). Then, this fragment was amplified from pJET1.2-*OsMac3*, fused with the maize codon-optimized *Cas9* (*zCas9*) from pKSE401 [62], and cloned into *Xba*I/*Sac*I-digested pKSE401 via Gibson Assembly (Gibson Assembly^®^ Master Mix, New England Biolabs, Ipswich, MA, USA) [63,64]. The resulting construct was named pKSEe401. Then, the *pAtUBQ10-DsRED1-NOSt* cassette was cloned into *Eco*RI-digested pKSEe401, resulting in pKSEe401R [61].

The plasmids for genome editing, pKSEe401R-*CsDRO1*, pKSEe401R-*CsDRO1-LIKE1*, and pKSEe401R-*CsDRO1-LIKE2* (Figure S8C,D), were constructed based on pKSEe401R. Target-specific *crispr*RNAs (crRNAs) were chosen using the CRISPOR and CRISPR-PLANT v.2 tools [65,66]. Three crRNAs were selected for each gene (Figure S8C). The absence of off-targets for crRNAs in the cucumber genome was checked using the BLASTN algorithm on the Cucurbit Genomics Database v.1 [48], with each crRNA sequence as query. The appropriate secondary structure of each of the resulting guide RNAs (gRNAs), containing a target-specific crRNA sequence and the conserved 80 bp *trans*-activating crRNA sequence, was checked using the RNAfold web server [67]. Cassettes containing three gRNAs for targeting each gene were cloned into the *Bsa*I-digested pKSEe401R vector using the Golden Gate protocol described by Xing et al. [62]. The absence of nucleotide rearrangements was verified by sequencing.

All binary vectors described in Section 4.3.1 and 4.3.2 were transferred in agrobacterial cells by electroporation [68]. The primer sequences for all applications are given in Table S1. The primers were designed using the Vector NTI Advance v.11.0 software (Thermo Fisher Scientific). Purified PCR primers were purchased from Evrogen (Moscow, Russia).

#### 4.3.3. Plant Transformation

The *Rhizobium* (*Agrobacterium*) *rhizogenes*-mediated plant transformation of cucumber seedlings was carried out as described previously [55,68,69,70].

#### 4.3.4. Preparation of Transgenic Cucumber Roots for Detection of CRISPR/Cas9-Mediated Mutations

Each transgenic root, harboring the T-DNA of the empty vector or the T-DNA of one of the three plasmids for genome editing, was divided into two parts. The first part of the root, 5–6 cm from the rot tip, was flash-frozen in liquid nitrogen followed by genomic DNA (gDNA) extraction. The second part of root with emerging lateral roots was divided into segments, with only one lateral root per segment, and fixed in a modified aldehyde-containing fixative [23,71] for 16–18 h at +4 °C, dehydrated in a graded ethanol series, and then stored in 70% ethanol at +4 °C until mutations of *CsDRO1*, *CsDRO1-LIKE1*, or *CsDRO1-LIKE2* via gene editing had been confirmed by DNA isolation, PCR, and sequencing.

gDNA was extracted from the control group (mixed root sample) and from each individual transgenic root (*CsDRO1*, *n* = 66; *CsDRO1-LIKE1*, *n* = 93; *CsDRO1-LIKE2*, *n* = 89) using Plant DNAzol (Thermo Fisher Scientific) [72] according to the manufacturer’s instructions. Each sequence of the three genes was PCR-amplified using the gDNA as a template. The primers (Table S1) were designed so that the region of CRISPR/Cas9 editing was within the resulting PCR product. Each PCR product was gel-separated, extracted, purified, and then analyzed using Sanger sequencing. After the nature of biallelic hetero- and homozygous mutations in *CsDRO1*, *CsDRO1-LIKE1*, or *CsDRO1-LIKE2* had been confirmed, the 3–5 segments with emerging lateral roots from each gene-edited transgenic root were used for lateral root measurements as described in Section 4.6.

### 4.4. Treatments with Exogenous Auxin

Five-day-old wild-type cucumber seedlings were incubated in aerated 1x Hoagland’s medium [73] supplemented with 10 μM of 1-naphthaleneacetic acid (NAA) (N0640, Sigma-Aldrich, Saint Louis, MO, USA) for 15 min, 30 min, 1 h, 2 h, and 6h, as described previously [70]. Root tips, 1 cm in length, were flash-frozen in liquid nitrogen. Each experiment included at least 37–45 seedlings and was repeated at least five times independently.

### 4.5. RT-qPCR Assays

Reverse transcription–quantitative polymerase chain reaction (RT-qPCR) assays were carried out as described previously [70]. The primers used for qPCR are listed in Table S1.

### 4.6. Fluorescence Protein Reporter Assays and Microscopy

The screening of transgenic roots in all experiments was performed based on the fluorescence of the DsRED1 reporter under a SteREO Lumar.V12 fluorescence stereomicroscope (Carl Zeiss, Oberkochen, Germany) with filter set 43 HE (EX BP 550/25, EM BP 605/70) [55,68,70].

For the localization of the mNeonGreen reporter, 7–9 mm long tips or upper segments of cucumber transgenic hairy roots harboring promoter–reporter fusions were fixed and sectioned as described previously [70]. For the estimation of the lateral root slope, segments of transgenic hairy roots with a confirmed knockout of individual *CsDROs* were fixed as explained in Section 4.3.4 and sectioned as described previously [70]. All microscopy procedures were performed as described previously [69,70]. In brief, the examination and imaging of fluorescent proteins and/or SR2200-stained cell walls were performed under an LSM 780 upright confocal laser scanning microscope (Carl Zeiss). The samples were imaged with a 488 nm excitation laser line for the mNeonGreen and/or the 405 nm excitation laser line and an emission spectrum of 412–464 nm for the SR2200-stained cell walls. The ZEN v2.3 pro software (Carl Zeiss) was used for image processing. To eliminate the autofluorescence and separate it from the mNeonGreen fluorescence, a Linear Spectral Unmixing Algorithm or Emission Fingerprinting with Lambda stacks in ZEN was used [69]. The lateral root slope angle was measured using the ZEN software after image acquisition.

### 4.7. Statistical Analyses

Plots for RT-qPCR and the lateral root slope angle were prepared using the R programming language, v. 4.0.2 [74], and the RStudio software, v. 1.3.1093 [75]. Code for the *boxplot* and *stripchart* functions from the base R package was used. The statistical analysis of the data was performed with Wilcoxon’s test from the base R package. Differences with *p*-values < 0.05 or < 0.01 were considered statistically significant. The RT-qPCR analysis of the relative expression levels of *CsDRO1*, *CsDRO1-LIKE1*, and *CsDRO1-LIKE2* under auxin treatment was performed with five biological replicates. At least 25 roots were used for each promoter–reporter fusion assay. The measurements of the lateral root slope angle were carried out on 3–6 lateral roots from each transgenic root with confirmed biallelic hetero- and homozygous mutations of *CsDRO1*, *CsDRO1-LIKE1*, or *CsDRO1-LIKE2* induced via gene editing.

## 5. Conclusions and Perspectives

The results of this study show that no individual *DRO1* homolog of cucumber affects the lateral root slope angle. However, further development of CRISPR-Cas9-mediated genome editing allows for the simultaneous knockout of multiple genes [76]. Based on this method, the simultaneous knockout of two or three cucumber *DRO1* homologs would permit analysis of their combined role in the control of the slope angle of lateral roots at the stage of their emergence from the parental root. The use of omics technologies [77] will also make it possible to identify the spectrum of targets of DRO1 homologs and to elucidate the real role of these genes/proteins in the establishment of the gravitropic response and in the formation of the slope angle of developing lateral roots, thereby allowing for the modification of root system architecture. Future research could explore additional genetic or environmental factors influencing the root architecture in cucumbers.

## Figures and Tables

**Table 1:** Similarity/Identity between Arabidopsis and cucumber DRO proteins.

Protein Names (Gene IDs of *C. sativus* cv Chinese Long v. 2)	Similarity (Identity) with *At*DRO1 Protein	Similarity (Identity) with *At*DRO2 Protein	Similarity (Identity) with *At*DRO3 Protein
*Cs*DRO1 (Csa7G448840)	63.5% (51.5%)	35.9% (27.4%)	64.5% (52%)
*Cs*DRO1-LIKE1 (CsDRO1L1, Csa1G597130)	43.7% (32.5%)	30.5% (23.9%)	43.5% (31.2%)
*Cs*DRO1-LIKE2 (CsDRO1L2, Csa3G645860)	25.7% (15%)	25.4% (16%)	27% (17%)

**Table 2:** Similarity/Identity between rice and cucumber DRO proteins.

Protein Names (Gene IDs of *C. sativus* cv Chinese Long v. 2)	Similarity (Identity) with *Os*DRO1 Protein	Similarity (Identity) with *Os*DRL1 Protein	Similarity (Identity) with *Os*DRL2 Protein	Similarity (Identity) with *Os*DRL3 Protein
*Cs*DRO1 (Csa7G448840)	51.7% (37.2%)	48.2% (36.2%)	31.5% (18.9%)	36.9% (29.4%)
*Cs*DRO1-LIKE1 (CsDRO1L1, Csa1G597130)	45.3% (32.5%)	43% (27%)	31% (20.4%)	32.1% (22.2%)
*Cs*DRO1-LIKE2 (CsDRO1L2, Csa3G645860)	22% (13%)	24% (13.2%)	24.3% (10.8%)	20.3% (9.3%)

**Table 3:** Cloning information of *CsDRO1*, *CsDRO1L1,* and *CsDRO1L2* promoter regions.

Gene Name	The Position of Cloning Region before the Predicted Translational Start Site	Size of Putative Promoter, bp
*CsDRO1*	from −3295 bp to −5 bp	3291
*CsDRO1L1*	from −808 bp to −1 bp	808
*CsDRO1L2*	from −2965 bp to −1 bp	2965

## Data Availability

All relevant data are within the paper and its Materials files. The plasmids pJET1.2-OsMac3 and pKSEe401R will be available at Addgene (www.addgene.org, accessed on 13 December 2023) and at WeKwikGene (wekwikgene.wllsb.edu.cn, accessed on 13 December 2023).

## Supplementary Materials

The following supporting information can be downloaded at https://www.mdpi.com/article/10.3390/ijms25041975/s1.

## Abbreviations

DRO1, DEEPER ROOTING 1; DRL/DRO1L, DEEPER ROOTING 1-LIKE; IAA, indole-3-acetic acid; NAA, 1–naphthaleneacetic acid; RT-qPCR, reverse transcription–quantitative polymerase chain reaction.
