# Supplementary material for: Do DEEPER ROOTING 1 Homologs Regulate the Lateral Root Slope Angle in Cucumber (Cucumis sativus)?
Source: Int J Mol Sci. 2024 Feb 6;25(4):1975. doi: 10.3390/ijms25041975 (PMC10888659; doi:10.3390/ijms25041975)
Supplement: Supplementary file 1 [file ijms-25-01975-s001.zip › ijms-2796432-supplementary.pdf]

International Journal of
Molecular Sciences

MDPI

Supplementary Materials

Do DEEPER ROOTING 1 homologs regulate the lateral root slope angle in cucumber (*Cucumis sativus*)?

Alexey S. Kiryushkin, Elena L. Ilina, Tatyana Y. Kiikova, Katharina Pawlowski and Kirill N. Demchenko

The following Supplementary Material is available for this article:

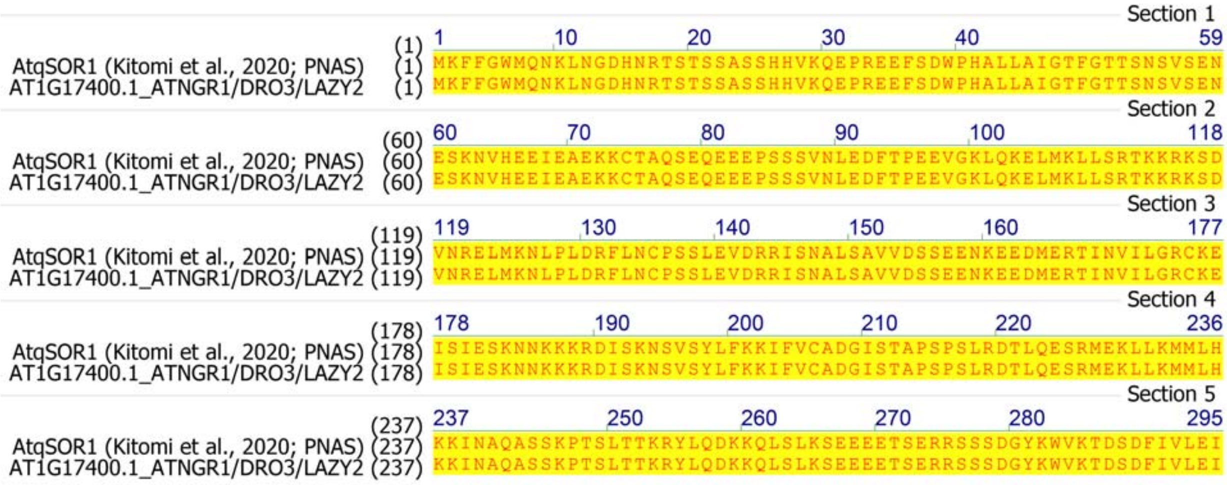

Figure S1. Alignment of *At*qSOR1 (Kitomi et al., 2020) and *At*NGR1/*At*DRO3/*At*LAZY2 (gene ID: AT1G17400.1) proteins. The sequences have 100% identity between each other.

Kitomi, Y., Hanzawa, E., Kuya, N., Inoue, H., Hara, N., Kawai, S., et al. (2020). Root angle modifications by the *DRO1* homolog improve rice yields in saline paddy fields. *Proc. Natl. Acad. Sci. USA* 117(35), 21242-21250. doi: 10.1073/pnas.2005911117

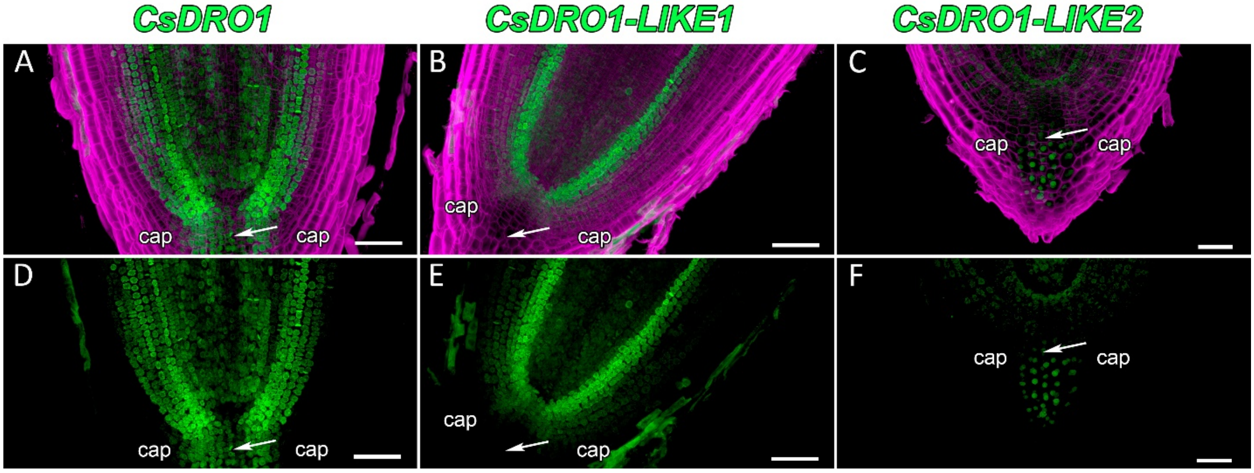

Figure S2. Localization of *CsDRO1*, *CsDRO1-LIKE1*, and *CsDRO1-LIKE2* expression in the root cap of *Cucumis sativus* (*pCsDRO::mNeonGreen-H2B)*. Confocal laser scanning microscopy of longitudinal vibratome sections. Expression of *CsDRO1* (**A**,**D**) was active in the root cap columella (white arrow). (**B**,**E**) Activity of *pCsDRO1L1* was not observed in the root cap columella (white arrow). (**C**,**F**) Expression of *CsDRO1L2* was maintained in the apical part of the root cap columella (white arrow). Green channel, fluorescence of mNeonGreen; magenta channel, SR2200-stained cell walls. Maximum intensity projection of z-series: of 12 optical sections, 8 μm in depth. Scale bars – (**A**,**B**,**D**,**E**), 100 μm; (**C**,**F**), 50 μm.

Figure S3. CsDRO1 (biallelic homozygous mutations)

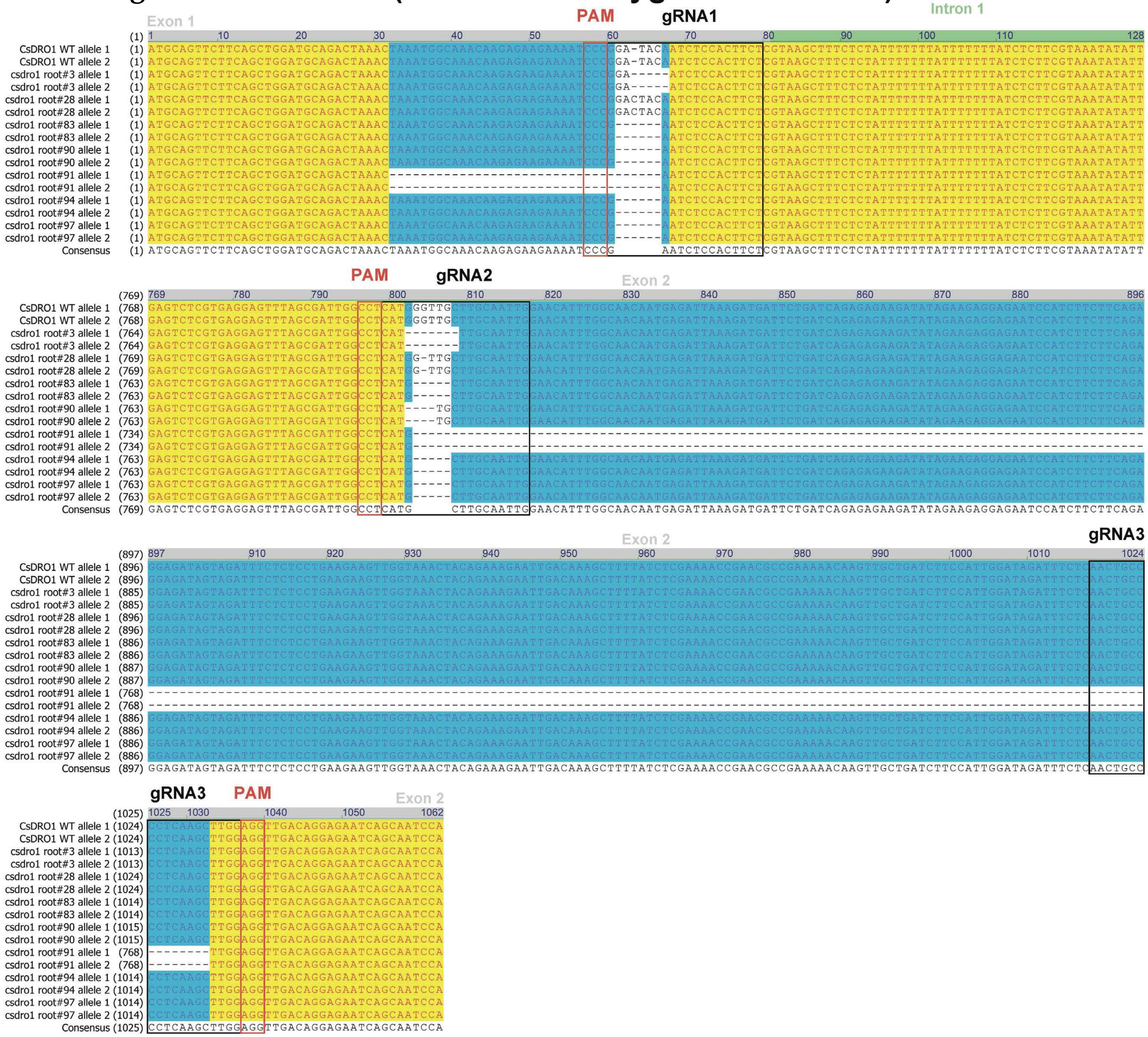

Figure S4. CsDRO1-LIKE1 (biallelic heterozygous mutations)

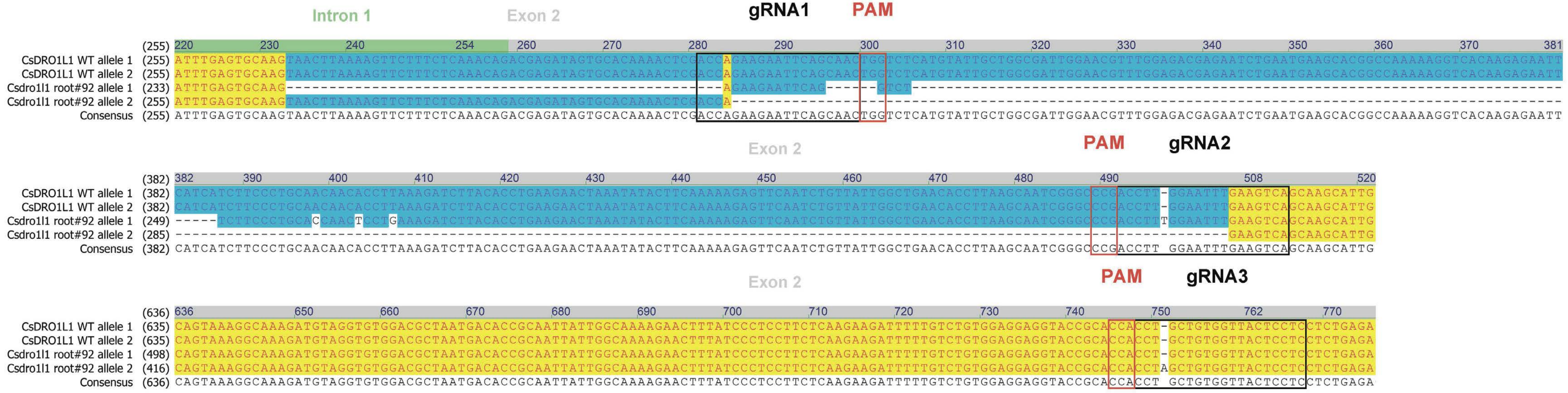

Figure S5. CsDRO1-LIKE1 (biallelic homozygous mutations)

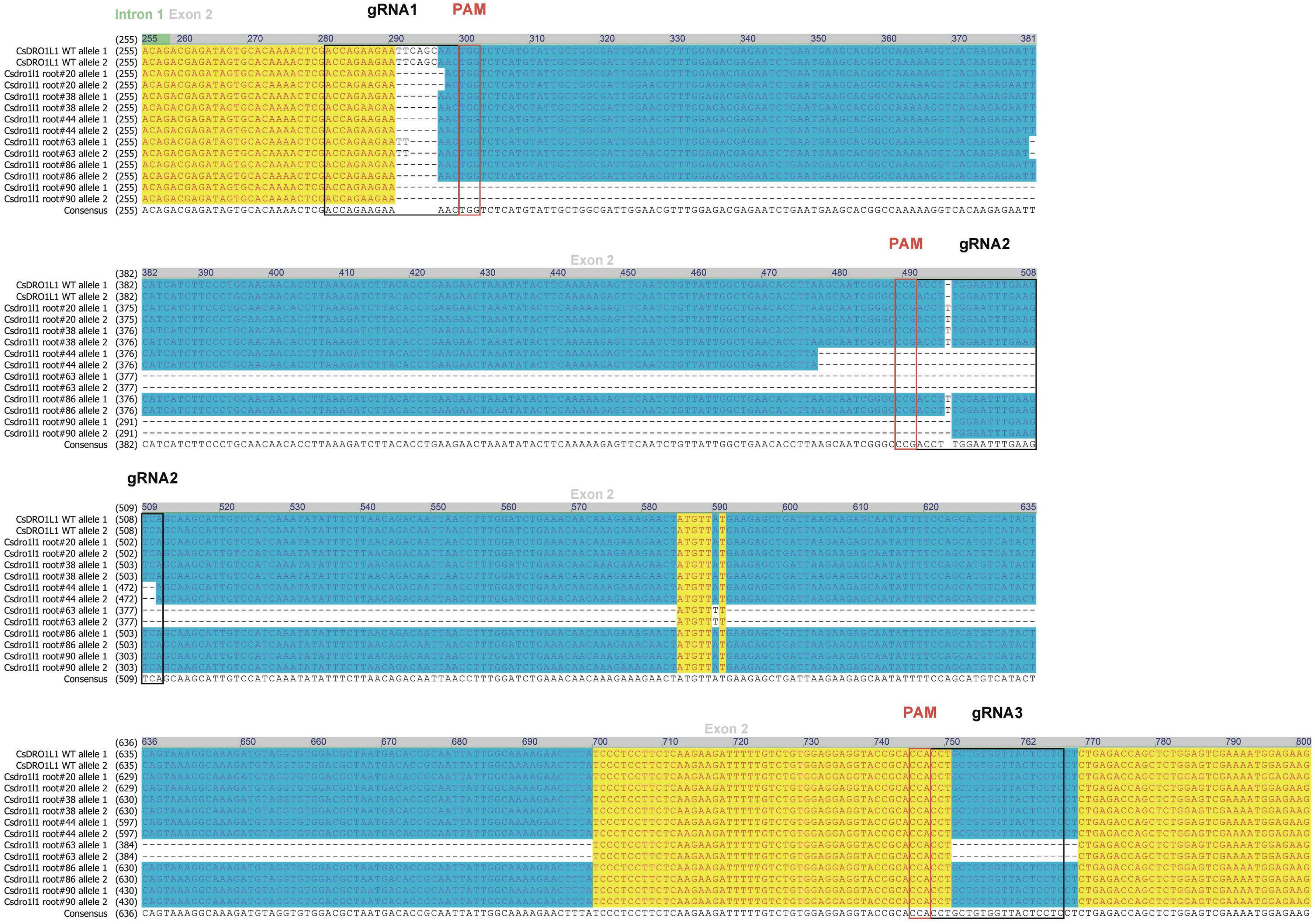

Figure S6. CsDRO1-LIKE2 (biallelic heterozygous mutations)

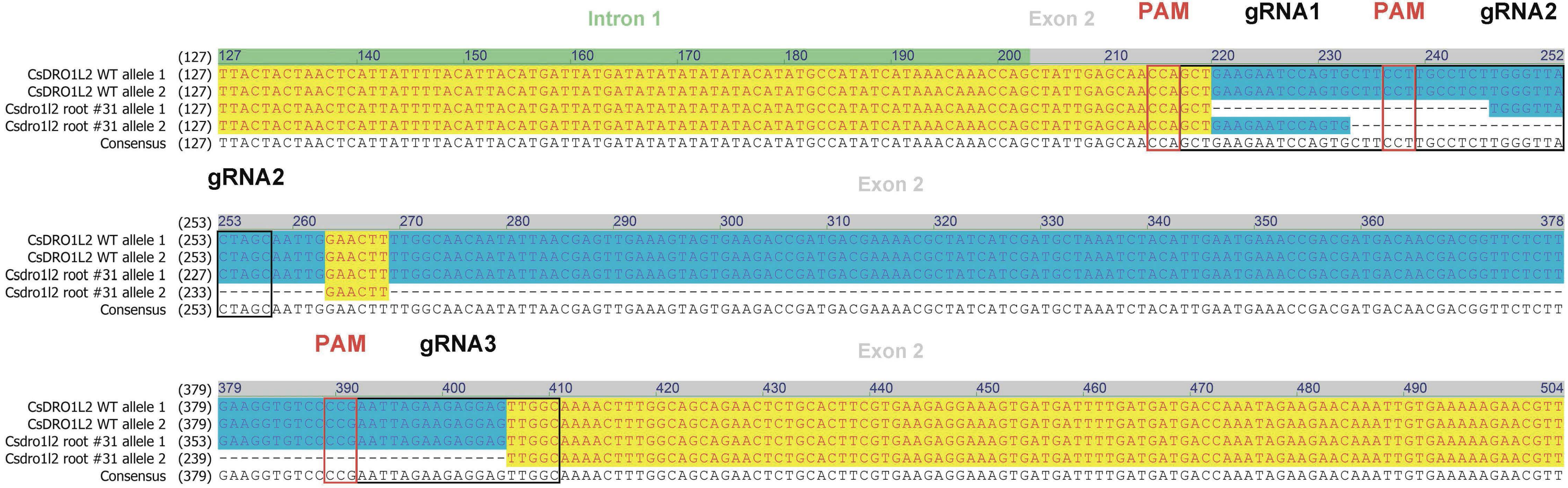

Figure S7. **CsDRO1-LIKE2 (biallelic homozygous mutations)**

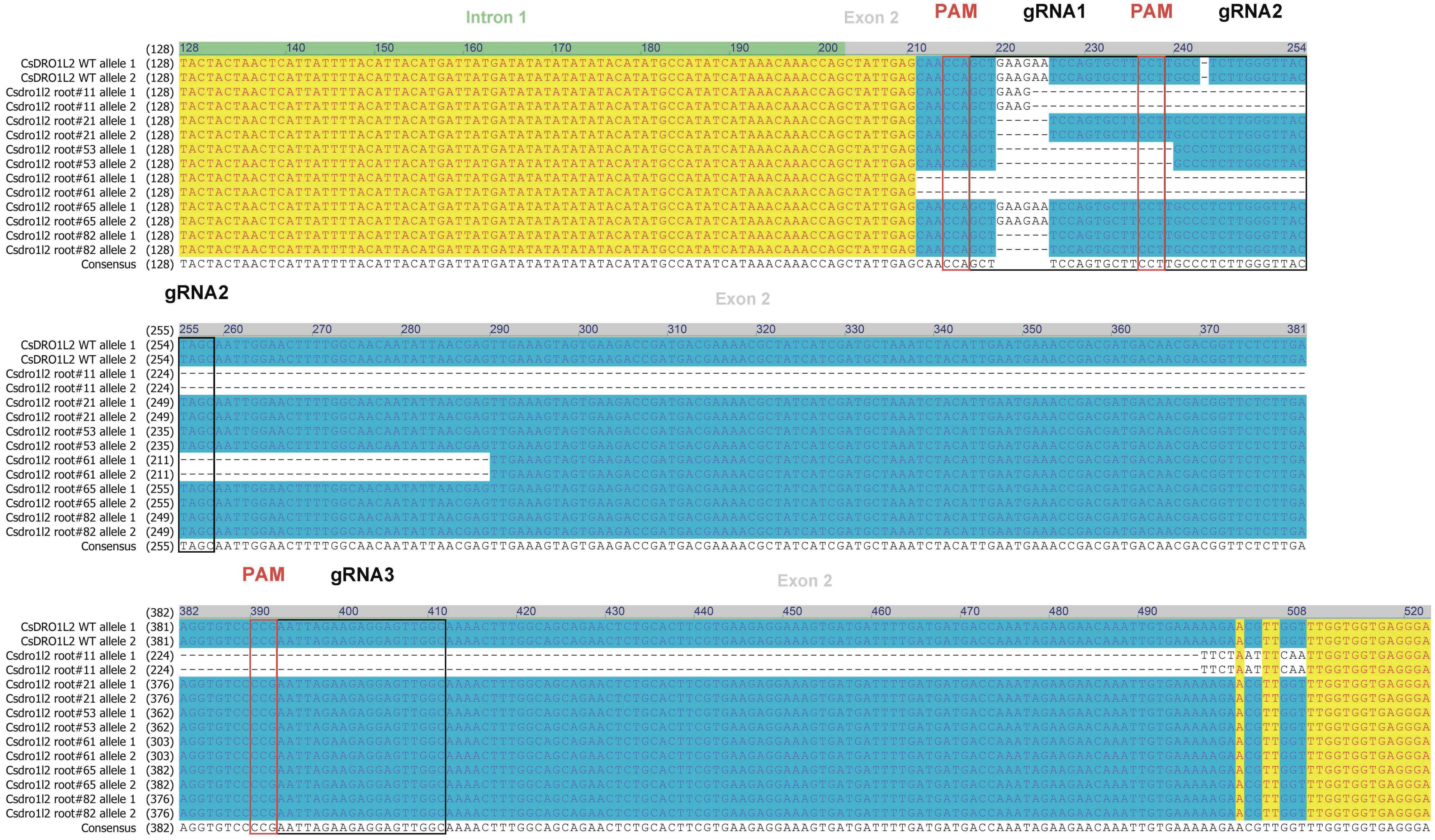

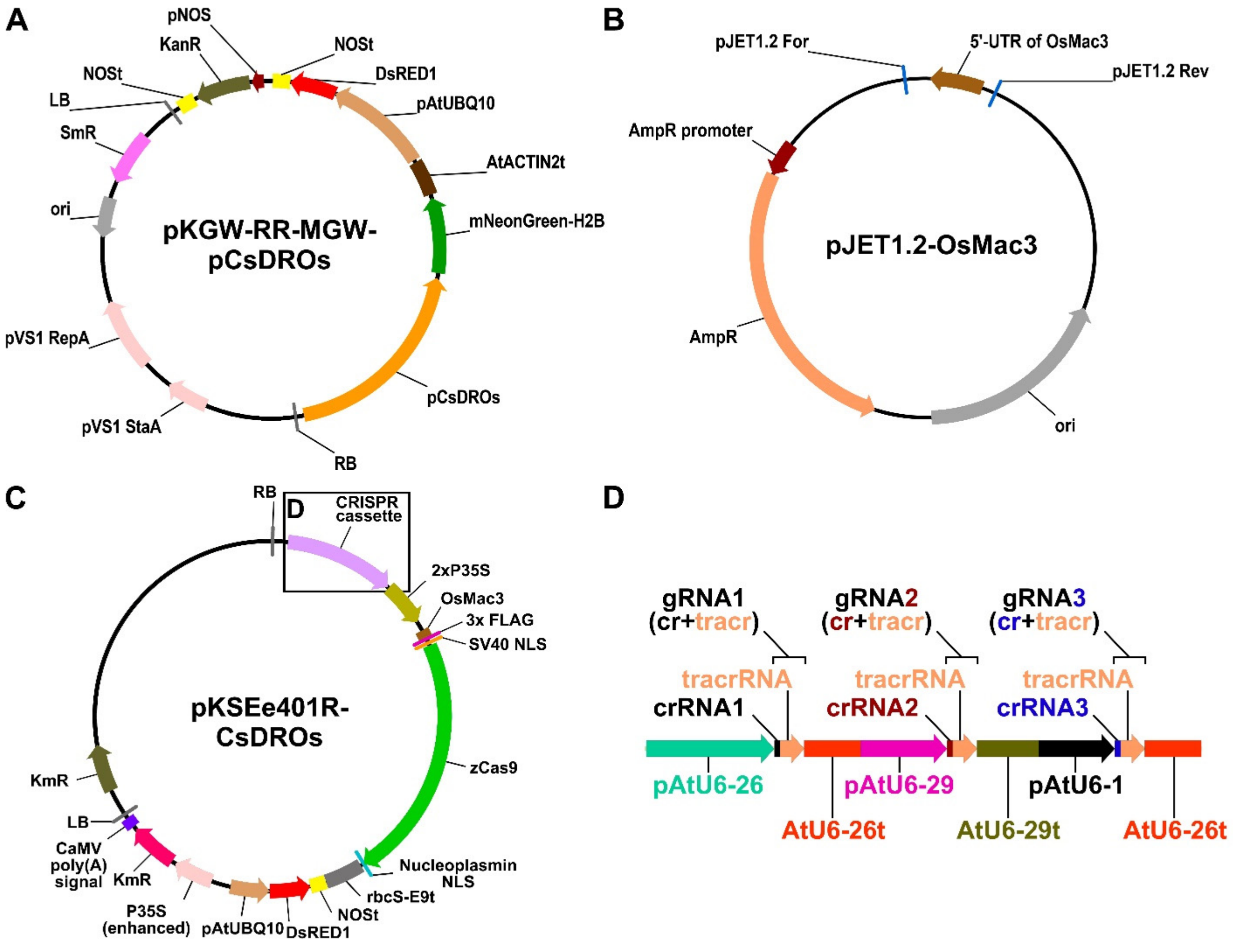

Figure S8. Maps of vectors used in this study. (**A**) Map of the pKGW-RR-MGW binary vector containing the *H2B-mNeonGreen* reporter under control of *CsDRO1*, *CsDRO1-LIKE1*, or *CsDRO1-LIKE2* promoter (*pCsDROs*). (**B**) Map of the pJET1.2 plasmid harboring the 158-bp 5′-UTR fragment of *OsMac3*. (**C**) Common map of the binary plasmids for genome editing, pKSEe401R-*CsDRO1*, pKSEe401R-*CsDRO1-LIKE1*, and pKSEe401R-*CsDRO1-LIKE2* (pKSEe401R-*CsDROs*). (**D**) General scheme of CRISPR cassette consisting of three guide RNA (gRNA)-expressing domains for targeting of each gene *CsDRO1*, *CsDRO1-LIKE1*, or *CsDRO1-LIKE2*. One domain comprises the promoter of the Arabidopsis *U6* small nucleolar RNA (*snoRNA*) gene (*pAtU6-26*, *pAtU6-29*, or *pAtU6-1*), the gRNA sequence (gRNA1, gRNA2, or gRNA3), and the Arabidopsis *U6 snoRNA* transcription terminator (*AtU6-26t* or *pAtU6-29t*). Each gRNA sequence, in turn, was designed to contain two fused sequences, the 19-bp target specific *crispr*RNA (crRNA1, crRNA2, or crRNA3) and the conservative 80-bp *trans*-activating crRNA (tracrRNA). Both pKGW-RR-MGW-*pCsDROs* (**A**) and pKSEe401R-*CsDROs* (**C**) binary vectors carried the *pAtUBQ10-DsRED1-NOSt* screening cassette (Limpens et al., 2004) in the backbone.

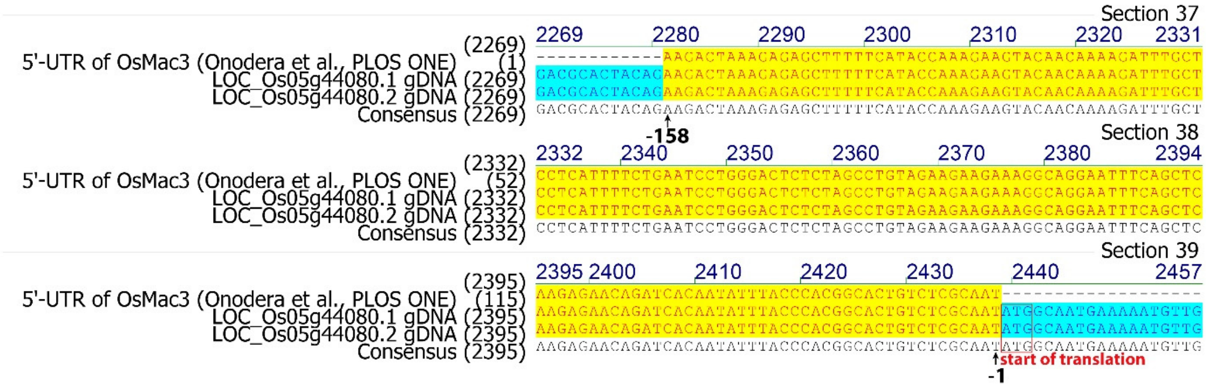

Figure S9. Fragment of the nucleotide sequence alignment between 5′-UTRs of LOC_Os05g44080.1, LOC_Os05g44080.2 (*Oryza sativa* v7.0 based on Phytozome v13), and 158-bp 5′-UTR fragment (from –158 to –1 b.p. before the ATG) of *OsMac3* from Onodera et al. (2018). The yellow color indicates the similar area between all sequences analyzed. Arrows with numbers indicate the position of the site relative to the translation start, ATG (marked with a red rectangle).

Limpens, E., Ramos, J., Franken, C., Raz, V., Compaan, B., Franssen, H., et al. (2004). RNA interference in *Agrobacterium rhizogenes*-transformed roots of *Arabidopsis* and *Medicago truncatula*. *J. Exp. Bot.* 55(399), 983-992. doi: 10.1093/jxb/erh122

Onodera, H., Shingu, S., Ohnuma, M., Horie, T., Kihira, M., Kusano, H., et al. (2018). Establishment of a conditional TALEN system using the translational enhancer dMac3 and an inducible promoter activated by glucocorticoid treatment to increase the frequency of targeted mutagenesis in plants. *PLOS ONE* 13(12), 1-13. doi: 10.1371/journal.pone.0208959

Table S1. List of primers used in this study.

Name	Sequence 5′-3′	Application
CsEF1a_qFor	ATGGGTAAGGAGAAGGTTCACATTAACATT	RT-qPCR analysis
CsEF1a_qRev	CGAACTTCCACAAAGCAATATCAATT	
CsDRO1_qFor	CCATAAAAAACAAGAGTCTCGTGAGGAG	
CsDRO1_qRev	GCTTTGTCAATTCTTTCTGTAGTTTACCAA	
CsDRO1-LIKE1_ qFor	GCAAAAGAACAACATCAAATTCAATCAA	
CsDRO1-LIKE1_ qRev	GTGCTTCATTCAGATTCTCGTCTCC	
CsDRO1-LIKE2_ qFor	GGATCATATAATTCTTCAAACCCTTCAAGT	
CsDRO1-LIKE2_ qRev	CAAGAGGCAAGGAAGCACTGGA	
pCsDRO1_For_*Xho*I	AA**CTCGAG**GCATATCAATGGCTCAAATCCAAAAG	Promoter cloning into pENTRattL4attR1_BSAI
pCsDRO1_Rev_*Kpn*I	AAA**GGTACC**TAAATGAAAAAAAGAGAATCAAAATCCCA	
pCsDRO1-LIKE1_For_*Xho*I	AA**CTCGAG**GAGATGTTCTTCCTCAAATCTTCTTCGT	
pCsDRO1-LIKE1_Rev_*Kpn*I	AAA**GGTACC**TTGTCAAAAGACGGATTGGCAAC	
pCsDRO1-LIKE2_For_*Xho*I	AA**CTCGAG**CTTGCTCAGCAACATCACGTATATCA	
pCsDRO1-LIKE2_Rev_*Kpn*I	AAA**GGTACC**CCAACTCAACAACTGAAACCCAAA	
OsMac3_For	AAGACTAAAGAGAGCTTTTTCATACCAAAG	Cloning of the *OsMac3* 5′-UTR fragment into pJET1.2 and sequencing
OsMac3_Rev	ATTGCGAGACAGTGCCGTGG	
OsMac3_For_Gi_*Xba*I	CATTTACGAACGATACTCGAGTAA**TCTAGA**AAGACTAAAGAGAGCTTTTTCATACCAAAG	Fusion *OsMac3* with *zCas9* and cloning into pKSE401 *via* Gibson Assembly
OsMac3_Rev_Gi	CGTGGTCCTTGTAATCCCCGTCGTGGTCCTTGTAATCCATATTGCGAGACAGTGCCGTGG	
zCas9_For_Gi	CAATATTTACCCACGGCACTGTCTCGCAATATGGATTACAAGGACCACGACGG	
zCas9_Rev_Gi_*Sac*I	ACCGATGATACGAACGAAAGCTCT**GAGCTC**TCACTTCTTCTTCTTCGCCTGCC	
zCas9_Rev1	CAACAGAGTTAGTCCCAATATCGAGG	Sequencing of the *OsMac3*-*zCas9* fusion after Gibson Assembly
zCas9_For2	ATGGATTACAAGGACCACGACGG	
zCas9_Rev2	CGAGACTTCGAGAGCCTAGCGGA	
zCas9_For3	CATCTGCGGAAGAAGCTCGTGG	
zCas9_Rev3	ATGAATCTGGTGTGGGATGCTGC	
zCas9_For4	CGATCAGTCGAAGAACGGCTACG	
zCas9_Rev4	CTCATTCTCCTCGTTGTCCAGGAAG	
zCas9_For5	ATCGTGGACCTCCTGTTCAAGACC	
zCas9_Rev5	CGTCGTAATCGCTGAGCCTGTTAATAT	
zCas9_For6	AAGCCTGAGAACATCGTCATTGAGAT	
zCas9_Rev6	TCCTCACATCGTAAACCTTGTAGTCCC	
zCas9_For7	GAAGGTCATCACCCTGAAGTCAAAGC	
zCas9_Rev7	GCCAGCGGAAGCCAGCA	
zCas9_For8	GGTTCGACAGCCCCACTGTG	
zCas9_Rev8	AGAGCTCTCACTTCTTCTTCTTCGCCTGCC	
pAtUBQ10_For_*Eco*RI	AA**GAATTC**CGGACGAAGTCCAGTAATAAACGG	Cloning of the *pAtUBQ10-DsRED1-NOSt* cassette
NOSt_Rev_*Eco*RI	AA**GAATTC**GATCTAGTAACATAGATGACACCGCGC	

Name	Sequence 5′-3′	Application
		into pKSEe401 and sequencing
M13_Rev	AACAGCTATGACCATG	Sequencing of the *pAtUBQ10-DsRED1-NOSt* cassette after insertion into pKSEe401
pAtUBQ10_Rev	TGTTAATCAGAAAAACTCAGATTAATCTACAAATT	
DsRED1_For	ATGGCGCGCTCCTCCAAG	
DsRED1_REV	CAGGAACAGGTGGTGGCGG	
CsDRO1_g1_DT1_*Bsa*I_F	ATATAT**GGTCTC**GATTGagaagtggagattgtatccGTT	Assembly of pKSEe401R-CsDRO1
CsDRO1_g1_DT1_F0	TGagaagtggagattgtatccGTTTTAGAGCTAGAAATAGC	
DT0_*Bsa*I_R2	ATATTATT**GGTCTC**AATCTCTTAGTCGACTCTACCAAT	
CsDRO1_g2_DT2_*Bsa*I_F2	ATATTATT**GGTCTC**AAGATTGcaattgcaagcaacccatgGTT	Assembly of pKSEe401R-CsDRO1
CsDRO1_g2_DT2_F0	TGcaattgcaagcaacccatgGTTTTAGAGCTAGAAATAGC	
CsDRO1_g3_DT3_R0	AACccaagcttgaggggcagttCAATCACTACTTCGTCTCTAACCAT	
CsDRO1_g3_DT3_*Bsa*I_R	ATTATT**GGTCTC**GAAACccaagcttgaggggcagttC	
CsDRO1-LIKE1_g1_DT1_*Bsa*I_F	ATATAT**GGTCTC**GATTGaccagaagaattcagcaacGTT	Assembly of pKSEe401R-CsDRO1-LIKE1
CsDRO1-LIKE1_g1_DT1_F0	TGaccagaagaattcagcaacGTTTTAGAGCTAGAAATAGC	
DT0_*Bsa*I_R2	the same as for the pKSEe401R-CsDRO1 assembly	
CsDRO1-LIKE1_g2_DT2_*Bsa*I_F2	ATATTATT**GGTCTC**AAGATTGtgacttcaaattccaaggtGTT	
CsDRO1-LIKE1_g2_DT2_F0	TGtgacttcaaattccaaggtGTTTTAGAGCTAGAAATAGC	
CsDRO1-LIKE1_g3_DT3_R0	AACcctgctgtggttactcctcCAATCACTACTTCGTCTCTAACCAT	
CsDRO1-LIKE1_g3_DT3_*Bsa*I_R	ATTATT**GGTCTC**GAAACcctgctgtggttactcctcC	
CsDRO1-LIKE2_g1_DT1_*Bsa*I_F	ATATAT**GGTCTC**GATTGaagcactggattcttcagcGTT	Assembly of pKSEe401R-CsDRO1-LIKE2
CsDRO1-LIKE2_g1_DT1_F0	TGaagcactggattcttcagcGTTTTAGAGCTAGAAATAGC	
DT0_*Bsa*I_R2	the same as for the pKSEe401R-CsDRO1 assembly	
CsDRO1-LIKE2_g2_DT2_*Bsa*I_F2	ATATTATT**GGTCTC**AAGATTGgctagtaacccaagaggcaGTT	
CsDRO1-LIKE2_g2_DT2_F0	TGgctagtaacccaagaggcaGTTTTAGAGCTAGAAATAGC	
CsDRO1-LIKE2_g3_DT3_R0	AACaattagaagaggagttggcCAATCACTACTTCGTCTCTAACCAT	
CsDRO1-LIKE2_g3_DT3_*Bsa*I_R	ATTATT**GGTCTC**GAAACaattagaagaggagttggcC	
M13_For	GTAAAACGACGGCCAGT	Sequencing of the CRISPR-cassettes in pKSEe401R-DRO1, - CsDRO1-LIKE1, and - CsDRO1-LIKE2
AtU6-26p_For	TGTCCCAGGATTAGAATGATTAGGC	
AtU6-29p_For	TTAATCCAAACTACTGCAGCCTGAC	
AtU6-29p_Rev	AGCCCTCTTCTTTCGATCCATCAAC	
AtU6-29t_For	CGTGTTTCAGCTCTCATGATCCTTG	
AtU6-1p_Rev	TATGCAAGTCTCACTCACACTCACG	
CsDRO1_5′-UTR _For	GTTCTGCTTCGGCATTCATCTTG	**PCR-amplification of the CsDRO1 ORF** and sequencing to search genome editing events within it
CsDRO1_3′-UTR _Rev	ATTTCAAAACAAGGGGTGGAGATTG	
CsDRO1_Intron1_For	TGTGCCTTGTTGCCTTGTTCC	Additional primers for sequencing to search genome editing events within the CsDRO1 ORF
CsDRO1_Intron2_Rev	TGATCTTTAGGATTTAATAATGAATATGCACA	
CsDRO1_Exon2_For	CTTTTATCTCGAAAACCGAACGCC	

Name	Sequence 5′-3′	Application
CsDRO1-LIKE1_5′-UTR _For	TCCCTGTTGCCAATCCGTCTTT	**PCR-amplification of the CsDRO1-LIKE1 ORF** and sequencing to search genome editing events within it
CsDRO1-LIKE1_3′-UTR _Rev	CCAAGCGAGAAAATAATGTCTTGTGAACT	
CsDRO1-LIKE2_5′-UTR _For	TTCTGAATAACCCCTTTAATTTTGGTG	**PCR-amplification of the CsDRO1-LIKE2 ORF** and sequencing to search genome editing events within it
CsDRO1-LIKE2_3′-UTR _Rev	CTATGGCAATAGTTACCTCTCATCTTCATC	
CsDRO1-LIKE2_Exon2_Rev1	TTCTAATTCGGGGACACCTTCAAG	Additional primers for sequencing to search genome editing events within the CsDRO1-LIKE2 ORF
CsDRO1-LIKE2_Exon2_For	TTGGAACTTTTGGCAACAATATTAACG	
CsDRO1-LIKE2_Exon2_Rev2	TTTGTTCTTCTATTTGGTCATCATCAAAGT	

Restriction enzyme sites in adaptors are underlined and given in **bold** style.

For long primers the primer sequences are separated from adaptors by the gray background.

The target specific *crispr*RNA sequences are given in small letters.
